# Host genotype and genetic diversity shape the evolution of a novel bacterial infection

**DOI:** 10.1038/s41396-021-00911-3

**Published:** 2021-02-18

**Authors:** Alice K. E. Ekroth, Michael Gerth, Emily J. Stevens, Suzanne A. Ford, Kayla C. King

**Affiliations:** 1 Department of Zoology, University of Oxford, Oxford, UK; 2 Department of Medical and Biological Sciences, Oxford Brookes University, Oxford, UK

**Keywords:** Molecular evolution, Bacterial evolution, Bacterial genetics

## Abstract

Pathogens continue to emerge from increased contact with novel host species. Whilst these hosts can represent distinct environments for pathogens, the impacts of host genetic background on how a pathogen evolves post-emergence are unclear. In a novel interaction, we experimentally evolved a pathogen (*Staphylococcus aureus*) in populations of wild nematodes (*Caenorhabditis elegans*) to test whether host genotype and genetic diversity affect pathogen evolution. After ten rounds of selection, we found that pathogen virulence evolved to vary across host genotypes, with differences in host metal ion acquisition detected as a possible driver of increased host exploitation. Diverse host populations selected for the highest levels of pathogen virulence, but infectivity was constrained, unlike in host monocultures. We hypothesise that population heterogeneity might pool together individuals that contribute disproportionately to the spread of infection or to enhanced virulence. The genomes of evolved populations were sequenced, and it was revealed that pathogens selected in distantly-related host genotypes diverged more than those in closely-related host genotypes. *S. aureus* nevertheless maintained a broad host range. Our study provides unique empirical insight into the evolutionary dynamics that could occur in other novel infections of wildlife and humans.

## Introduction

Emerging infectious diseases have led to serious declines in wildlife populations. Heavy population losses have been documented in rabbits following the introduction of Myxoma virus [1], amphibians from Chytrid fungus [2, 3], Tasmanian devils from Devil Facial Tumour Disease [4] and brown bats from *Pseudogymnoascus destructans* fungus [5]. Emergence events can result from reservoir host spill-overs [6], jumps between host species [1], the evolution of a new pathogen trait that allows for exploitation of a new host [7] or by invading a new environment [8, 9]. These novel interactions can be initially harmful [10] or entirely avirulent [11] to the host, and pathogen virulence and replication rates can evolve [12].

Pathogen adaptation can play a role in emergence [13, 14]. Thus, the need to understand novel pathogen evolution in its new host population is central to predicting and managing the consequences. It has been shown that pathogen evolution can be shaped by many factors in established host–pathogen relationships, such as host genetic diversity [15], spatial structure [16] and gene flow [17–22]. Of particular interest is the role of host genotype and genetic diversity. Although genetically homogeneous populations are generally rare in the wild, many conservation efforts focus on declining, isolated and island populations often with low genetic diversity [4, 23]. There are also growing numbers of homogeneous populations being managed for agriculture [24, 25]. Are these populations hotbeds for increasingly damaging, emerging infections?

Host genotype and diversity can affect pathogen evolution in many ways. Individual host genotypes can vary in their immune-based resistance to infection [26, 27] but also in other aspects that might impact upon infection success, such as pathogen avoidance [28, 29] and starvation responses [30]. Specialisation, or a narrow variance in infectiousness across different hosts [31], can evolve when pathogens infect homogeneous host populations of single genotypes [21, 32]. An explanation is that pathogen populations acquire mutations that are neutral [21] or beneficial [33] in the focal host environment but costly in others. The ability for pathogens to specialise suggests that host genotypes can represent distinct selective environments. At the population level, high host genetic diversity can increase the odds of pathogens encountering resistant genotypes. This outcome has been shown to limit disease spread [23, 34–39], virulence evolution [20], evolutionary rates [40] and also impede parasite adaptation [41] resulting in host range expansion [21, 22, 42] in established interactions.

It is commonly assumed that genetically diverse host populations contain individuals that have protective immunity [36] (also in some theoretical models predicting pathogen emergence [39]). However, when pathogens are newly-introduced, most individuals could be susceptible [1, 4]. Resistance may not spread until well after emergence when some pathogen evolution will have already taken place [12]. Yates et al. [14] found that if pathogen adaptation takes place during emergence, diverse host populations can have a small positive effect given they can contain individuals contributing greatly to transmission (i.e. super-spreaders). It thus remains unclear whether host genetic diversity has an impact on novel pathogen evolution early on in the association when most hosts are susceptible.

In this study, we investigated whether host genotype and population-level genetic diversity drive evolutionary changes in the virulence, infectivity and host range of a newly-introduced pathogen. We passaged pathogenic *Staphylococcus aureus* between populations of a novel host species—the nematode *Caenorhabditis elegans*—which varied in genotype (24 natural isolates in monoculture) and diversity (polyculture of all 24 isolates). The nematode hosts used were randomly selected across a wild isolate *C. elegans* phylogeny [43] to represent a diverse spectrum of genetic backgrounds (Table S1). *C. elegans* are likely exposed to *Staphylococcus* spp. in natural environments, such as compost [44] and on button mushrooms [45] where these nematodes are vectors of bacteria causing blotch disease [46]. However, *S. aureus* per se has not been found to date to be a natural, established pathogen of *C. elegans*. Pathogenic *S. aureus* strains are known to jump regularly between host species, including a range of domestic animals, wild hosts, such as rodents, non-human primates and bats [47–49]. After a period of evolution, we compared the trajectories of each evolved population across sympatric host genotypes, as well as between host monocultures and polycultures. We measured changes in pathogen-induced host mortality and infection load as these traits relate to *S*. *aureus* virulence and infectivity [50, 51], respectively. We additionally compared the evolutionary trajectory of host range across treatments by evaluating these traits of evolved populations on a novel host genotype. Our main predictions were that (a) pathogens selected in a host genotype would increase their virulence and infectivity on that genotype, (b) pathogens selected in diverse host polycultures would be more constrained in their evolution of virulence and infectivity and (c) pathogens selected in a host genotype would specialise and show reduced virulence/infectivity on novel hosts. The molecular basis of the adaptive process, and its relationship to host genetic distance, were also explored.

## Materials and methods

### Nematode hosts and maintenance

Twenty-three *Caenorhabditis elegans* wild genotypes were randomly selected across the phylogenetic tree presented in Andersen et al. [43] (Table S1). The lab-adapted N2 genotype was added to the collection. Nematode populations were kept evolutionarily static throughout experiments. Stock populations of all nematode genotypes were maintained at 20 °C on nematode growth medium (NGM) plates seeded with their food, 600 μL *Escherichia coli* OP50 in Luria-Bertani Broth.

To synchronise the life stages of all 24 nematode genotypes, populations were treated with a 1:1 solution of NaClO and 5M sodium hydroxide. This solution kills bacterial cells and nematodes, leaving only unhatched eggs. Eggs were subsequently incubated at 20 °C on a shaking platform overnight and then plated out onto *E. coli* seeded NGM plates. Once synchronised, L4 stage nematodes were washed off using 2 mL M9 buffer supplemented with 10% Triton X-100, and added on the top of 1000 μL filter-tips placed within Eppendorf tubes. Nematodes were then washed three times using 500 μL M9 and centrifuged for 1 min at 755 × *g* to remove any bacteria lining the nematode cuticle.

### Bacterial strain and stock preparation

Nematode populations were exposed to *Staphylococcus aureus* MSSA476 (GenBank: BX571857.1), a human pathogen isolate, sourced from the University of Liverpool [52]. Bacterial infection in the nematode gut occurs upon ingestion. This pathogen lyses the cells lining the nematode gut wall [29] and ultimately causes host mortality [53]. A randomly-selected colony was inoculated into 20 mL Tryptone Soy broth (TSB) and grown for 24 h with shaking at 220 rpm at 30 °C. This single ancestral stock population was used for all experiments and frozen in a 1:1 ratio of sample to 60% glycerol at −80 °C.

### Evolution experiment

We passaged pathogens across ten host generations through (i) 24 biological replicates of monoculture nematode host populations (each replicate was comprised of a different host genotype), and (ii) six polyculture replicate host populations of all 24 genotypes combined. We also had six no-host control replicates of pathogen passaging to account for genomic changes that occurred outside the host. The passage experiment was performed in 24-well plates containing 500 μL 1.1% viscous media [54] and 100 μL of concentrated *S. aureus* inoculum per well to mitigate pathogen avoidance [55], digging [56] and aggregating [57, 58] nematode behaviours, and therefore to ensure equal pathogen exposure. The viscous media solution consisted of TSB media supplemented with 2.3 g HPMC cellulose (Hydroxypropyl methyl cellulose, Sigma-Aldrich). Populations of ~500 L4s in each exposure well were incubated at 25 °C for 24 h. All animals, alive and dead, were removed from the culture 24 h after pathogen exposure for pathogen extraction and passage. The nematodes were placed onto 1000 μL filters within 1.5 mL tubes and centrifuged at 2656 × *g* for 2 min. The supernatant was removed, and nematodes were re-suspended in 500 μL M9 and further centrifuged at 755 × *g* for 2 min. This washing process was repeated three times. To harvest pathogens to passage, 0.7 mm Zirconia beads (BioSpec products) were added to a resuspension of 10% nematode population sample with 100 μL M9, and placed in a mini beadbeater (BeadBug, Microtube homogeniser) at 320 rpm for 45 s. The crushed solution was plated onto Mannitol Salt Agar (MSA) to select *S. aureus*, and incubated for 24 h at 37 °C. Approximately 100 colonies per replicate were picked and grown in 5 mL TSB overnight, shaking at 220 rpm at 30 °C. This pathogen has been shown previously to adapt to treatment conditions using this approach [50, 59]. Host populations were then exposed using these cultures in the next passage (see Supplementary Fig. 1).

### Host killing and infection load

We examined the effects of host genotype and genetic diversity on pathogen-induced host mortality and infection load, at the point of introduction and after pathogen evolution. For both ancestral and evolved pathogen populations, we compared these two traits across host genotypes in monoculture, and between host monocultures and polycultures. A limitation of our assays is our inability to account for pathogen phenotypic adaptation to laboratory protocols specifically.

Approximately 200 nematodes per replicate population were incubated at 25 °C for 24 h in 250 μL viscous media supplemented with 50 μL pathogen. Nematodes were considered dead when they did not respond to the touch of a platinum wire [60]. Host mortality remained at ~1–4% across all treatments in our experimental set-up. Because no death was observed in the food control, we considered *S. aureus* to be a harmful infectious agent to *C.elegans*, as found previously on other media types [51].

To measure infection load, we randomly picked and washed in three 10 μL droplets of M9, and crushed ten nematodes per replicate. Pathogen load was estimated by counting colony forming units (cfu) of 100-fold diluted *S. aureus* grown overnight at 37 °C on MSA plates. These assays were repeated five times.

We tested whether pathogens evolved to specialise on their sympatric host population. A random selection of pathogen populations selected within five single-host genotypes and five polyculture replicates were exposed to their sympatric host population and a population comprised of the novel genotype, CB4857. Exposures were performed in the same manner as above, with three technical replicates. Infection loads were estimated from five worms per replicate. Degree of pathogen specialisation was calculated by subtracting pathogen performance (pathogen-induced host mortality, infection load) in the novel host from the that in the sympatric host population [41, 61].

### Bacterial genome extraction and analysis

Forty clones per replicate population from the ancestor and passage 10 were grown separately overnight in 200 μL TSB at 30 °C at 150 rpm. Clones were pooled together per replicate into 1.2 mL cultures, and DNA was extracted following the DNeasy Blood & Tissue Kit (Qiagen) protocol. DNA purification then followed the DNeasy Blood and Tissue spin-column protocol (Qiagen). Purity and quality of extracted DNA was assessed using the Nanodrop ND-100 and Qubit Fluorometer. DNA was normalised to a concentration of around 50 ng/μL in 10 mM Tris-Cl and sent to the Oxford Genomics Centre at the Wellcome Centre for Human Genetics where paired-end libraries were prepared and sequenced as 150 bp run on an HiSeq4000 (Illumina).

Fastq read files were checked for quality and trimmed using fastp 0.19.6 using default options. Reads were then aligned to the *S. aureus* MSSA476 reference genome sequence (NCBI accession number, GCF_000011525.1) using the bwa 0.7.17 ‘mem’ algorithm [62] and the option ‘-M’ to ensure single best alignment. Samtools version 1.9 was then used to filter out PCR duplicates and to remove singletons. The Genome Analysis Toolkit v.4.0.11.0 (GATK) Haplotype caller module was used to detect variants with the options ‘--sample-ploidy 40 --heterozygosity 0.0001 --indel-heterozygosity 0.00001’ as previously described [50]. With this ploidy level, we had an average coverage of 322.85*x* ± 39.78, allowing for a 2.5% SNP frequency in a single clone. Variants were then hard-filtered using standard practices as described before [50]. All variants present in the evolved *S. aureus* lines that were also present in the sequenced ancestral strain and the no-host control were removed before further analyses. Evolutionary distances between *S. aureus* population and time points were determined by calculating the Euclidian distances between variant frequencies.

To account for the impact of host genetic distance on pathogen evolution in our experiments, we determined the genetic diversity between host monocultures. We first downloaded the hard-filtered variants of *C. elegans* from the ‘Caenorhabditis elegans Natural Diversity Resource’ (https://www.elegansvariation.org/) and extracted all fixed variants (AF = 1) associated with any of the 24 monocultures.

### Statistical analysis

All data analysis was carried out in R v 3.6.0. Parametric tests were used in cases where the data met the required assumptions, otherwise equivalent non-parametric tests were used. Assumptions of normality of data were confirmed with Shapiro tests, and equality of variances with F-tests (or Levene’s test in cases of comparisons among >2 groups). Data point outliers were confirmed using the Dixon test and removed where appropriate. Multiple comparisons of infection load and host mortality data were corrected for using the FDR method for generating *p* values.

We assessed evolutionary changes in pathogen populations selected in host genotypes by using Binomial General Linear Models and one-way Analysis of Variance (ANOVA) to examine variation in host mortality and infection load, respectively, across host genotypes. We used Kendall’s rank correlation to account for tied pairs when making multiple correlations between non-parametric infection load and host mortality data between ancestral and evolved pathogen populations. We performed Spearman’s rank correlations to determine if infection load and pathogen-induced host mortality were associated across host genotypes for evolved *S. aureus*.

We used Split-plot ANOVAs to compare host mortality and infection load between host monoculture to polyculture treatments. This approach accounted for within-treatment replicate variation and time-point (ancestral vs. evolved pathogen) of our pairwise genetic comparison data. We conversely used a two-sample *t* test to compare ancestral to evolved pathogen infection loads in host polycultures as these data met the assumption of equal variances.

To determine if *S. aureus* evolved to host specialism over time, we compared the degree of host range (calculated by subtracting pathogen traits in novel from sympatric host associations) to 0 using Wilcoxon-rank sum tests on non-normal data and one sample *t* tests on normally distributed data. We compared the host range between pathogen populations selected within monocultures and polycultures using Wilcoxon rank-sum tests.

## Results

### Infection at point of pathogen introduction

**Fig. 1:**
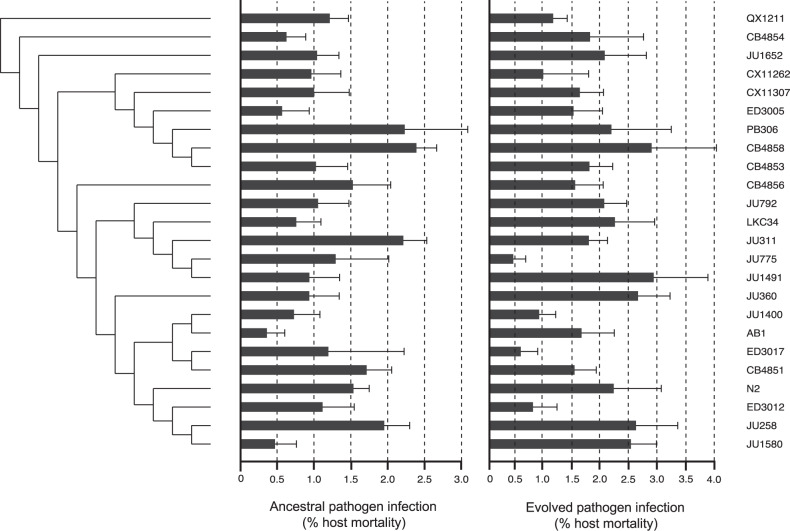
Phylogeny of the 24 *C. elegans* wild genotypes used in the experiment mapped against the virulent impacts of ancestral and their sympatric, evolved pathogen populations (measured by mean % host mortality caused by infection ± SE). Host mortality for each host isolate was measured across five replicates. The tree is rooted by the most genetically disparate host isolate (QX1121), see Andersen et al. [43] for full description.

Previous studies have found that wild *C. elegans* genotypes differed in susceptibility to infections of *Bacillus thruringiensis* [27] and *Serratia marcescens* [20, 63] implying a shared coevolutionary history or significant standing genetic variation for resistance [64]. Although *C. elegans* may encounter *Staphylococcus* spp. on the microbiota of vegetation [45], human-acquired *S. aureus* is unlikely to naturally co-occur in the same environment. This likely novelty of the interaction may explain the lack of significant host genetic variation in resistance, as measured by pathogen-induced host mortality (Fig. 1, Table 1) and infection load (Fig. S2, Table 1) across 24 host genotypes at the beginning of the experiment. This pattern held when accounting for the genetic distance between host genotypes (*p* > 0.05). It is possible there is host genetic variation for other aspects of fitness during *S. aureus* infection (e.g. host offspring number, population growth) not measured herein. Infection load was also not associated with pathogen virulence across host genotypes (Kendall’s rank correlation, *z* = 0.036, tau = 0.002, *p* = 0.97).

**Table 1:** Extent of variation in pathogen killing ability across host genotypes upon introduction and after experimental evolution.

	d.f.	Test statistic	*p*
Host mortality			
Ancestor	23	*X*^2^ = 28.738	0.19
Evolved	23	*X*^2^ = 39.875	**0.016**
Infection load			
Ancestor	23	*F* = 0.38	0.99
Evolved	23	*F* = 0.56	0.94

Statistically significant *p*-values are in bold.

### Extent of pathogen evolution within host genotypes

After 10 bouts of selection, we found that pathogen virulence and infection load evolved differently across host genotypes. When evolved pathogens were tested on their sympatric host genotypes and compared, pathogen-induced host mortality was significantly variable across all host backgrounds (Fig. 1, Table 1). To understand how host genotypes could select for different virulence levels, we conducted a GO-term analysis (Supplementary Methods) comparing the putative functions of genes that differ in sequence between host genotypes. We found that in 8 out of 10 pairwise comparisons (Table S2), host genotypes that favoured divergent levels of pathogen virulence differed in functions relating to metal ion binding. Only 3 of 10 comparisons of host genotypes with pathogens causing comparable levels of virulence (Table S2) showed evidence of differences in metal ion binding. The function of metal binding is an important host factor in infectious disease outcomes as changes in host sequestering of metal binding proteins (such as zinc, sodium and magnesium,) could affect bacterial uptake of nutrients essential for bacterial growth [65, 66] and influence disease severity.

**Fig. 2:**
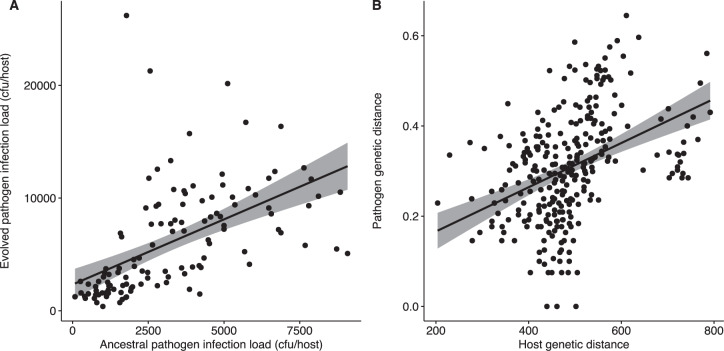
Pathogen evolution across host genotypes. **A** Correlation between within-host infection load of ancestral and evolved *S. aureus* in monoculture. **B** Correlation between pairwise genetic distance of host genotypes and evolved sympatric pathogens. Genetic distance was measured by calculating the Euclidean distances between variant frequencies of each host genotype or pathogen population.

In contrast, infection load did not evolve to vary significantly across host genotypes (Tables 1,  S2) even taking into account host genetic distance (*p* > 0.05). Infection loads in ancestral and sympatric host genotypes were positively associated (Fig. 2A; Kendall’s rank correlation, *z* = 8.49, tau = 0.53, *p* < 0.001) revealing an escalation of infectivity within sympatric host genotypes.

We compared the evolutionary trajectories of both pathogen traits to determine if similar selective forces were acting on them throughout the experiment. No correlation emerged between infection load and pathogen-induced host mortality within sympatric host genotypes after pathogen evolution (Spearman’s rank correlation, *s* = 269590, rho = 0.015, *p* = 0.87), suggesting that these traits evolved independently.

We examined whether the patterns of pathogen molecular evolution reflected genetic similarities among the 24 host genotypes in monoculture. We calculated pairwise genetic distances between all 24 evolved *S. aureus* monoculture populations using SNP differences (see Methods). We then mapped the pathogen genetic distances against those of the corresponding nematode genotypes. We found that host and pathogen genetic distances were positively correlated after ten passages (Fig. 2B; Mantel test, *r* = 0.41, *p* = 0.035). We conclude that significant genetic differentiation has been generated among pathogens replicating in different host genotypes, and that closely-related host genotypes might represent similar selective environments to pathogens.

### Limited infection load and higher virulence in diverse host populations

**Fig. 3:**
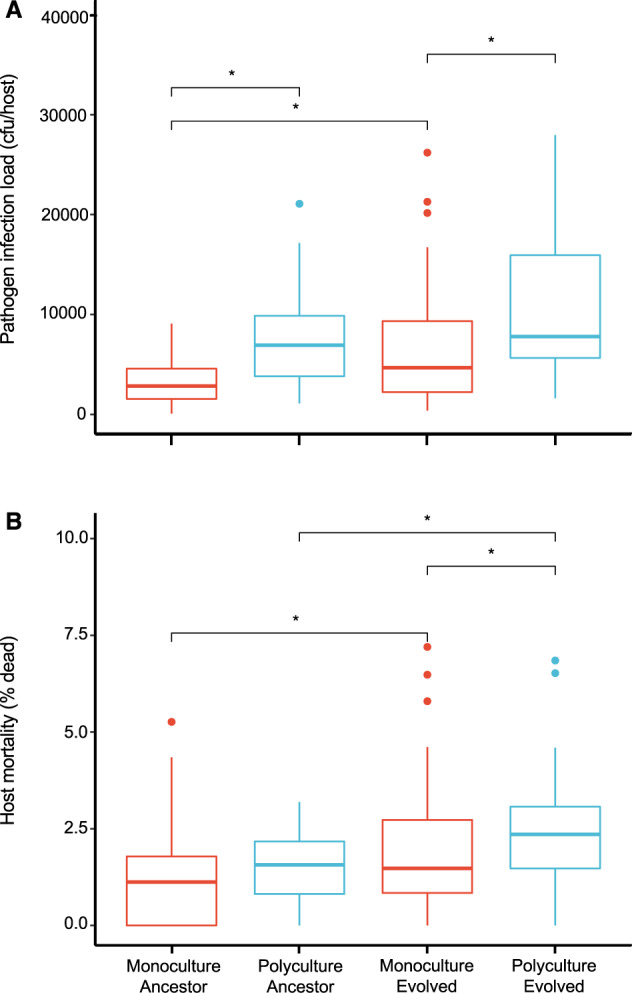
Pathogen evolution and host genetic diversity. Changes in (**A**) infection load (cfu/host) and (**B**) pathogen-induced mortality in host monocultures (red) and polycultures (blue). Monocultures consisted of 24 biological replicates of different host genotypes and polycultures of six replicates of pools of all 24 genotypes. Five technical replicates for all assays. Asterisks show significant comparisons (*p* < 0.05).

We compared within-host infection loads in monoculture and polyculture host populations. We found that infection loads of ancestral (Fig. 3A, Table 2) pathogens were both higher in host polycultures compared to in host monocultures. Infection load increased linearly between the ancestral pathogen and the evolved pathogen in sympatric host genotypes in monoculture, but evolved loads were the same as ancestral loads in polycultures (Fig. 3A, Table 2). This finding suggests that, unlike in monocultures, the pathogen could not increase its infectivity in highly diverse populations.

**Table 2:** Host population type as a predictor of variation in pathogen traits upon introduction and after experimental evolution.

	d.f.	Test statistic	*p*
Host mortality			
* Monoculture*_*anc*_ *vs. monoculture*_*evo*_		*W* = 5508	**0.006**
* Polyculture*_*anc*_ *vs. polyculture*_*evo*_		*W* = 278	**0.02**
* Monoculture*_*anc*_ *vs. polyculture*_*anc*_	27	*F* = 0.67	0.41
* Monoculture*_*evo*_ *vs. polyculture*_*evo*_	27	*F* = 21.99	**<0.001**
Infection load			
* Monoculture*_*anc*_ *vs. monoculture*_*evo*_		*W* = 4662	**<0.001**
* Polyculture*_*anc*_ *vs. polyculture*_*evo*_	58	*t* = −1.91	0.062
* Monoculture*_*anc*_ *vs. polyculture*_*anc*_	27	*F* = 26.83	**<0.001**
* Monoculture*_*evo*_ *vs. polyculture*_*evo*_	27	*F* = 17.59	**<0.001**

Statistically significant *p*-values are in bold.

Pathogen virulence increased linearly over time in both host monocultures and polyculture populations. We found that ancestral pathogens killed the same proportions of hosts in monocultures and polycultures, but pathogens became more harmful to both population types over evolutionary time (Fig. 3B, Table 2). Peak pathogen virulence was greater after evolution in host polycultures where *S. aureus* killed a greater proportion of host overall (Fig. 3B, Table 2).

We attempted to evaluate the molecular basis for the differences in the evolution of pathogen traits between host monocultures and polycultures. After identifying SNPs, we grouped the putative functions of the corresponding genes in relation to virulence, metabolism, and adherence (Supplementary Methods, Table S3). We examined the degree to which these functions were under selection by comparing within-population SNP frequencies between evolving pathogens in monocultures and polycultures. We found that SNPs in genes with putative functions related to siderophore activity increased in frequency in monocultures (Fig. S3). These molecules are considered a public good that facilitate more iron extraction from hosts and increased pathogen growth [67, 68], and are positively associated with host killing in nematodes [67]. Although we observed an increase in in vitro siderophore production by all evolved pathogens compared to the ancestor, these changes were not significant between host treatments (Fig. S4) and not related to host mortality in monocultures or polycultures (Fig. S4). Other aspects of siderophore activity in vivo may have been altered, but not captured by our in vitro measure.

To determine whether the pathogen populations selected within host monocultures and polycultures evolved at different rates, we measured the genetic distance of evolved pathogen populations from the ancestor in both treatments. We found genomic divergence in both sets of pathogen populations, but host monocultures and polycultures drove the same rates of pathogen evolution on average (Fig. S5).

### Maintenance of broad host range in evolved pathogens

**Fig. 4:**
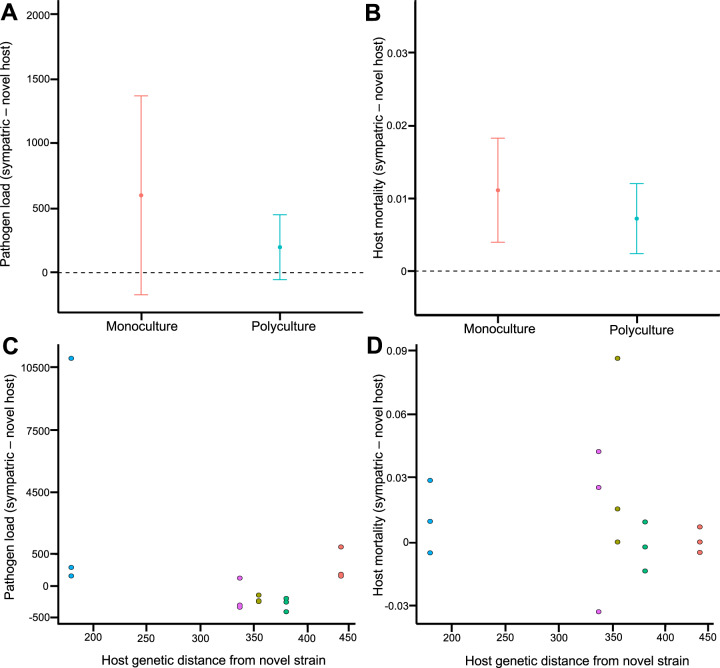
Maintenance of broad host range in evolved pathogens. Measurements of pathogen performance in host population genetic backgrounds were taken for (**A**) pathogen infection load (cfu/host) and (**B**) host mortality (% proportion of dead in population) in five replicate populations of monoculture-evolved (Red) and polyculture-evolved (Blue) *S. aureus*. (**C**) Infection load and (**D**) pathogen-induced host mortality were not a function of genetic distance between sympatric and novel host genotypes. Host genetic distances were calculated by measuring the Euclidean distances between isolates. Degree of specialism was measured by subtracting the infection metrics in novel (CB4857) hosts from those in sympatric hosts (Kawecki and Ebert [61], Morley et al. [41]), where by 0 (dotted line) shows no difference and a broad host range. The novel host genotype was used for both monoculture and polyculture comparisons. Points show mean ± SE of five technical replicates for (**A**, **B**) and three for (**C**, **D**).

We assessed whether pathogen populations selected in host genotypes in monoculture evolved a narrower host range than those selected in host polycultures. For pathogen populations selected in host genotypes, we compared the pathogen-induced host mortality and infection load on their sympatric and a novel host genotype. We did not find any loss or gain of virulence or infectivity (Fig. 4A, B, Table 3). We further examined whether genetic distance between sympatric and novel host genotypes was a factor in pathogen performance. There was a trend, albeit insignificant, towards a negative association between host genetic distance and infection load (Fig. 4C; Pearson’s product moment correlation, *r* = −0.49, d.f. = 13, *p* = 0.06), but no relationship with host mortality (Fig. 4D; Pearson’s product moment correlation, *r* = −0.10, d.f. = 13, *p* = 0.72). These results demonstrate that these pathogens do not constrict their host range during evolution within a host genotype in monoculture. We also found that pathogens evolved in host polycultures similarly maintained their generalist host range and were able to equally kill and colonise the novel host genotype (Fig. 4A, B, Table 3). Maintenance of the broad host range seems to be a conserved outcome of pathogen evolution in this study regardless of the host genotype or level of population genetic diversity.

**Table 3:** Pathogen selection treatment as a predictor of specificity of host adaptation. Pathogen traits were compared between evolved pathogen populations in their sympatric and novel host (CB4857) populations.

	d.f.	Test statistic	*p*
Host mortality			
Monocultures	14	*t* = 1.55	0.14
Polycultures		*V* = 67	0.14
Monocultures vs. polycultures		*W* = 111	0.97
Infection load			
Monocultures		*V* = 52	0.68
Polycultures		*V* = 85.5	0.16
Monocultures vs. polycultures		*W* = 141	0.25

## Discussion

The dynamics and outcomes of pathogen evolution are understudied in most emerging disease systems [69]. Here, we directly tested the selective impact of host genotype and genetic diversity on pathogen virulence and infectivity by tracking their evolution in a novel nematode-bacteria interaction. Throughout the experiment, we found that virulence and infectivity predominately increased, but evolved independently across the range of host genetic backgrounds. More specifically, host killing ability and infection load were not significantly associated at the point of emergence, after evolution, or within host genotypes or diverse host populations. Contrasting evolutionary trajectories between pathogen traits have been found after the initial spread of infection in wild, emerging disease systems [12] and weak associations between traits are recorded in human infectious diseases [70]. This larger pattern across the study suggests that different sources or strengths of selection acted on pathogen populations infecting host genotypes and diverse pools of nematodes.

We found that higher pathogen virulence evolved, on average, in both single-host genotype monocultures and diverse host polycultures. Our selection regime, whereby pathogens were passaged in a series of naïve hosts still living at the time of pathogen extraction, can account for some of the increase. In many serial passage experiments, the costs of virulence and rapid replication are removed favouring a rise in host harm [71, 72]. However, significant variation in virulence arose among host genotypes in monoculture. Host metal ion binding was a common distinction in the gene functions of host genotypes that selected for disparate levels of pathogen virulence. Otherwise known as ‘nutritional immunity’ [66], hosts can retain metal ions as a strategy for preventing infection. Whilst this might hamper bacterial growth [66, 73], fewer metals in the host environment might impose selection on pathogens to increase host metal exploitation and virulence. We did not find higher siderophore production (iron-scavenging molecules) in vitro in the more virulent pathogen populations, but *S. aureus* can extract host metals, including iron, via other molecular mechanisms [74, 75]. Metal ion binding is essential to staphylococci as a component of numerous biochemical processes, including energy production, DNA synthesis, and defence against oxidative stress [66]. It is also important in the regulation of virulence factors, whereby staphylococcal cells that successfully sequester metal ions from a host exhibit greater pathogenicity [66].

The evolution of higher virulence within genetically diverse host populations, beyond that observed in host monocultures, is contrary to previous findings that host heterogeneity reduces pathogen virulence [20]. We hypothesise that pooling 24 host genotypes concentrated hosts able to retain/give up metals—and selected for higher virulence—and/or hosts better able to transmit pathogens. This latter scenario of super-spreading, suggested by Yates et al. [14] as a potential consequence of host population diversity, could allow for more local transmission within each passage in viscous media. Theory predicts that the virulence of emerging pathogens is predicted to be larger when transmission is high as the pathogen can spread more easily amongst susceptible hosts [76]. This positive relationship between transmission and virulence has been shown to favour increased virulence in novel infections in nature [77].

Several studies have shown that host population diversity can limit parasite success [34, 78, 79], even that of emerging pathogens [34, 80, 81], whilst host homogeneity should confer susceptibility. Although this prediction was not realised in the patterns of virulence evolution in this study, we found that pathogen evolution in host monocultures yielded higher infection loads. There was an overall escalation of infectivity within sympatric host genotypes. We conversely found that infection load did not evolve to increase within genetically diverse host populations. Despite the limitation on evolution, the infection loads in these populations were initially higher, on average, than those in host monocultures. This outcome may possibly result from the ecological consequences of population diversity. If resource competition increases with the relatedness in host populations, as predicted by the niche partitioning theory [82], *C. elegans* stress pathways could be up-regulated and strengthen their defence mechanisms [83] in monocultures compared to diverse populations. Alternatively, as stated earlier, we also hypothesise that diverse populations might have experienced more within-culture pathogen transmission. Whether this increased transmission might facilitate increased infectivity and virulence by this pathogen remains to be tested. Aspects of population variation beyond immune-based resistance could be vital to pathogen colonisation.

We observed that pathogen populations diverged at the genomic level, and more so following selection in distantly-related host genotypes. This result suggests that host genetic distance corresponds with a difference in selection environments, an assumption sometimes made in predicting the success of host species jumps [84, 85]. In the context of host species jumps [84], host phylogenetic relatedness can determine infection success upon a host switch [84]—as with primate lentivirus [86], rabies virus [87] and *Drosophila* RNA viruses [85]—although closely-related species may both be susceptible despite the distance due to the loss or gain of immune functions associated with the pathogen [84, 88]. Despite genomic divergence, pathogen populations selected in monocultures of host genotypes did not evolve a contracted host range compared to those in diverse host polycultures. Perhaps the nematode genotypes used were not sufficiently different to favour specialisation, and resistance must spread before specialisation can occur. Or the experimental period was not long enough. However, it has been predicted that a narrower host range should arise in pathogens infecting homogeneous host populations due to faster fixing of beneficial alleles, with slower evolution occurring in diverse host populations [40]. We found that the host population types drove the same speed of pathogen genomic evolution and SNPs remained at relatively low frequencies in all pathogen populations. Moreover, Gibson et al. [21] previously found that after 20 nematode host generations and the existence of substantial genetic variation in host resistance, the established pathogen *Serratia marcescens* did not consistently evolve specialism. The maintenance of a broad host genotype range in our study reflects the generalist strategies of other emerging infections [24, 89, 90]. Generalist pathogens are more likely to emerge than specialist pathogens [31, 91]. Through high mutation rates, generalist pathogens can produce diverse genetic variants [91, 92] helping them avoid host immune responses and limiting specialised host resistance. Not evolving to specialise on a host genotype can also be explained by the natural history of *S. aureus*, a prevalent host-shifter [93, 94]. This bacterium has a large host range [47, 95–98] and has emerged in domesticated bovine [95], poultry [99], and rabbits [97], as well as invertebrate species, including *C. elegans* [29, 100].

This study reveals that any changes in pathogen traits and genomes following introduction can be variable across host genotypes and levels of population genetic diversity. As anthropogenic alterations to habitats and geographic ranges increase opportunities for contact between novel pathogens and naive hosts, we should expect more infectious disease emergence [37]. Increased knowledge on the evolution of novel infectious disease can provide insight on managing the harm caused by pathogens in human medicine, wildlife conservation and agriculture—and on mitigating their spread [101]. Our results suggest that host differences in metal-sequestration, as well as the ecological implications of host population diversity, warrant further consideration as drivers of infection outcomes over evolutionary time in recent wildlife and human pathogens.

## Supplementary information

Supplemental material

## Data Availability

Raw read data for the bacterial genomic sequences are available on the NCBI website under accession number PRJNA685531. The experimental data can be accessed on the Dryad repository (10.5061/dryad.w0vt4b8q4).

## References

[1] Kerr PJ (2012). Myxomatosis in Australia and Europe: a model for emerging infectious diseases. Antivir Res.

[2] Daszak P, Berger L, Cunningham AA, Hyatt AD, Green DE, Speare R (1999). Emerging infectious diseases and amphibian population declines. Emerg Infect Dis.

[3] Stuart SN, Chanson JS, Cox NA, Young BE, Rodrigues ASL, Fischman DL (2004). Status and trends of amphibian declines and extinctions worldwide. Science.

[4] Hawkins CE, Baars C, Hesterman H, Hocking GJ, Jones ME, Lazenby B (2006). Emerging disease and population decline of an island endemic, the Tasmanian devil *Sarcophilus harrisii*. Biol Conserv.

[5] Leopardi S, Blake D, Puechmaille SJ (2015). White-nose syndrome fungus introduced from Europe to North America. Curr Biol.

[6] Gascoyne SC, Laurenson MK, Lelo S, Borner M (1993). Rabies in African wild dogs (*Lycaon pictus*) in the Serengeti region, Tanzania. J Wildl Dis.

[7] Shortridge KF, Zhou NN, Guan Y, Gao P, Ito T, Kawaoka Y (1998). Characterization of Avian H5N1 Influenza Viruses from Poultry in Hong Kong. Virology.

[8] Kurtenbach K, Hanincová K, Tsao JI, Margos G, Fish D, Ogden NH (2006). Fundamental processes in the evolutionary ecology of *Lyme borreliosis*. Nat Rev Microbiol.

[9] Engering A, Hogerwerf L, Slingenbergh J (2013). Pathogen-host-environment interplay and disease emergence. Emerg Microbes Infect.

[10] Alizon S, Hurford A, Mideo N, Baalen MV (2008). Virulence evolution and the trade-off hypothesis: history, current state of affairs and the future. J Evol Biol.

[11] Gandon S, Hochberg ME, Holt RD, Day T (2013). What limits the evolutionary emergence of pathogens?. Philos Trans R Soc B Biol Sci.

[12] Tardy L, Giraudeau M, Hill GE, McGraw KJ, Bonneaud C (2019). Contrasting evolution of virulence and replication rate in an emerging bacterial pathogen. Proc Natl Acad Sci USA.

[13] Antia R, Regoes RR, Koella JC, Bergstrom CT (2003). The role of evolution in the emergence of infectious diseases. Nature.

[14] Yates A, Antia R, Regoes RR (2006). How do pathogen evolution and host heterogeneity interact in disease emergence?. Proc R Soc B.

[15] Regoes RR, Nowak MA, Bonhoeffer S (2000). Evolution of virulence in a heterogeneous host population. Evolution.

[16] Boots M, Mealor M (2007). Local interactions select for lower pathogen infectivity. Science.

[17] Lion S, Gandon S (2015). Evolution of spatially structured host–parasite interactions. J Evol Biol.

[18] Chin KM, Wolfe MS (1984). Selection on *Erysiphe graminis* in pure and mixed stands of barley. Plant Pathol.

[19] Thrall PH, Burdon JJ (2003). Evolution of virulence in a plant host-pathogen metapopulation. Science.

[20] White PS, Choi A, Pandey R, Menezes A, Penley M, Gibson AK (2020). Host heterogeneity mitigates virulence evolution. Biol Lett.

[21] Gibson AK, Baffoe‐Bonnie H, Penley MJ, Lin J, Owens R, Khalid A (2020). The evolution of parasite host range in heterogeneous host populations. J Evol Biol.

[22] Bono LM, Gensel CL, Pfennig DW, Burch CL (2012). Competition and the origins of novelty: experimental evolution of niche-width expansion in a virus. Biol Lett.

[23] King KC, Lively CM (2012). Does genetic diversity limit disease spread in natural host populations?. Heredity.

[24] Daszak P (2000). Emerging infectious diseases of wildlife—threats to biodiversity and human health. Science.

[25] Manley R, Boots M, Wilfert L (2015). REVIEW: emerging viral disease risk to pollinating insects: ecological, evolutionary and anthropogenic factors. J Appl Ecol.

[26] Carius HJ, Little TJ, Ebert D (2001). Genetic variation in a host-parasite association: potential for coevolution and frequency-dependent selection. Evolution.

[27] Schulenburg H, Müller S (2004). Natural variation in the response of *Caenorhabditis elegans* towards *Bacillus thuringiensis*. Parasitology.

[28] Chang HC, Paek J, Kim DH (2011). Natural polymorphisms in *C. elegans* HECW-1 E3 ligase affect pathogen avoidance behaviour. Nature.

[29] Gravato‐Nobre MJ, Hodgkin J (2005). *Caenorhabditis elegans* as a model for innate immunity to pathogens. Cell Microbiol.

[30] Webster AK, Hung A, Moore BT, Guzman R, Jordan JM, Kaplan REW (2019). Population selection and sequencing of *Caenorhabditis elegans* wild isolates identifies a region on chromosome III affecting starvation resistance. G3 (Bethesda).

[31] Leggett HC, Buckling A, Long GH, Boots M (2013). Generalism and the evolution of parasite virulence. Trends Eco Evol.

[32] Ebert D (1998). Experimental evolution of parasites. Science.

[33] Kubinak JL, Ruff JS, Hyzer CW, Slev PR, Potts WK (2012). Experimental viral evolution to specific host MHC genotypes reveals fitness and virulence trade-offs in alternative MHC types. Proc Natl Acad Sci USA.

[34] van Houte S, van, Ekroth AKE, Broniewski JM, Chabas H, Ashby B, Bondy-Denomy J (2016). The diversity-generating benefits of a prokaryotic adaptive immune system. Nature.

[35] Lively CM (2010). The effect of host genetic diversity on disease spread. Am Nat.

[36] Ekroth AKE, Rafaluk-Mohr C, King KC (2019). Host genetic diversity limits parasite success beyond agricultural systems: a meta-analysis. Proc R Soc B.

[37] Altizer S, Harvell D, Friedle E (2003). Rapid evolutionary dynamics and disease threats to biodiversity. Trends Eco Evol.

[38] Dwyer G, Elkinton JS, Buonaccorsi JP (1997). Host heterogeneity in susceptibility and disease dynamics: tests of a mathematical model. Am Nat.

[39] Chabas H, Lion S, Nicot A, Meaden S, Houte S, van, Moineau S (2018). Evolutionary emergence of infectious diseases in heterogeneous host populations. PLOS Biol.

[40] González R, Butković A, Elena SF. Role of host genetic diversity for susceptibility-to-infection in the evolution of virulence of a plant virus. Virus Evol. 2019;5. 10.1093/ve/vez024. 10.1093/ve/vez024 PMC6863064 31768264

[41] Morley D, Broniewski JM, Westra ER, Buckling A, van Houte S (2016). Host diversity limits the evolution of parasite local adaptation. Mol Ecol.

[42] Coffey LL, Vignuzzi M (2011). Host alternation of chikungunya virus increases fitness while restricting population diversity and adaptability to novel selective pressures. J Virol.

[43] Andersen EC, Gerke JP, Shapiro JA, Crissman JR, Ghosh R, Bloom JS (2012). Chromosome-scale selective sweeps shape *Caenorhabditis elegans* genomic diversity. Nat Genet.

[44] Montalvo-Katz S, Huang H, Appel MD, Berg M, Shapira M (2013). Association with soil bacteria enhances p38-dependent infection resistance in *Caenorhabditis elegans*. Infect Immun.

[45] Rossouw W, Korsten L (2017). Cultivable microbiome of fresh white button mushrooms. Lett Appl Microbiol.

[46] Grewal PS (1991). Relative contribution of nematodes (*Caenorhabditis elegans*) and bacteria towards the disruption of flushing patterns and losses in yield and quality of mushrooms (*Agaricus bisporus*). Ann Appl Biol.

[47] Mrochen DM, Schulz D, Fischer S, Jeske K, El Gohary H, Reil D (2018). Wild rodents and shrews are natural hosts of *Staphylococcus aureus*. Int J Med Microbiol.

[48] Peton V, Le Loir Y (2014). *Staphylococcus aureus* in veterinary medicine. Infect Genet Evol.

[49] Schaumburg F, Mugisha L, Peck B, Becker K, Gillespie TR, Peters G (2012). Drug-resistant human *Staphylococcus Aureus* in sanctuary apes pose a threat to endangered wild ape populations. Am J Primatol.

[50] Ford SA, Kao D, Williams D, King KC (2016). Microbe-mediated host defence drives the evolution of reduced pathogen virulence. Nat Commun.

[51] King KC, Brockhurst MA, Vasieva O, Paterson S, Betts A, Ford SA (2016). Rapid evolution of microbe-mediated protection against pathogens in a worm host. ISME J.

[52] Holden MTG, Feil EJ, Lindsay JA, Peacock SJ, Day NPJ, Enright MC (2004). Complete genomes of two clinical *Staphylococcus aureus* strains: Evidence for the rapid evolution of virulence and drug resistance. Proc Natl Acad Sci USA.

[53] Sifri CD, Begun J, Ausubel FM, Calderwood SB (2003). *Caenorhabditis elegans* as a model host for *Staphylococcus aureus* pathogenesis. Infect Immun.

[54] Papkou A, Guzella T, Yang W, Koepper S, Pees B, Schalkowski R (2019). The genomic basis of Red Queen dynamics during rapid reciprocal host–pathogen coevolution. Proc Natl Acad Sci USA.

[55] Pradel E, Zhang Y, Pujol N, Matsuyama T, Bargmann CI, Ewbank JJ (2007). Detection and avoidance of a natural product from the pathogenic bacterium *Serratia marcescens* by *Caenorhabditis elegans*. Proc Natl Acad Sci USA.

[56] Andersen EC, Bloom JS, Gerke JP, Kruglyak L (2014). A variant in the neuropeptide receptor npr-1 is a major determinant of *Caenorhabditis elegans* growth and physiology. PLOS Genet.

[57] Hodgkin J, Doniach T (1997). Natural variation and copulatory plug formation in *Caenorhabditis elegans*. Genetics.

[58] de Bono M, Bargmann CI (1998). Natural variation in a neuropeptide Y receptor homolog modifies social behavior and food response in *C. elegans*. Cell.

[59] Ford SA, Williams D, Paterson S, King KC (2017). Co-evolutionary dynamics between a defensive microbe and a pathogen driven by fluctuating selection. Mol Ecol.

[60] Garsin DA, Sifri CD, Mylonakis E, Qin X, Singh KV, Murray BE (2001). A simple model host for identifying Gram-positive virulence factors. Proc Natl Acad Sci USA.

[61] Kawecki TJ, Ebert D (2004). Conceptual issues in local adaptation. Ecol Lett.

[62] Li H, Durbin R (2009). Fast and accurate short read alignment with Burrows–Wheeler transform. Bioinformatics.

[63] Schulenburg H, Ewbank JJ (2004). Diversity and specificity in the interaction between *Caenorhabditis elegans* and the pathogen *Serratia marcescens*. BMC Evol Biol.

[64] Duxbury EM, Day JP, Maria Vespasiani D, Thüringer Y, Tolosana I, Smith SC (2019). Host-pathogen coevolution increases genetic variation in susceptibility to infection. eLife.

[65] Corbin BD, Seeley EH, Raab A, Feldmann J, Miller MR, Torres VJ (2008). Metal chelation and inhibition of bacterial growth in tissue abscesses. Science.

[66] Cassat JE, Skaar EP (2012). Metal ion acquisition in *Staphylococcus aureus*: overcoming nutritional immunity. Semin Immunopathol.

[67] Dale SE, Doherty-Kirby A, Lajoie G, Heinrichs DE (2004). Role of siderophore biosynthesis in virulence of *Staphylococcus aureus*: Identification and characterization of genes involved in production of a siderophore. Infect Immun.

[68] West SA, Griffin AS, Gardner A, Diggle SP (2006). Social evolution theory for microorganisms. Nat Rev Microbiol.

[69] Bolker BM, Nanda A, Shah D (2010). Transient virulence of emerging pathogens. J R Soc Interface.

[70] Leggett HC, Cornwallis CK, Buckling A, West SA (2017). Growth rate, transmission mode and virulence in human pathogens. Philos Trans R Soc B Biol Sci.

[71] Rafaluk C, Jansen G, Schulenburg H, Joop G (2015). When experimental selection for virulence leads to loss of virulence. Trends Parasitol.

[72] Day T (2001). Parasite transmission modes and the evolution of virulence. Evolution.

[73] Sigel A, Sigel H. Metal ions in biological systems. New York: CRC Press; 1998.

[74] Becker KW, Skaar EP (2014). Metal limitation and toxicity at the interface between host and pathogen. FEMS Microbiol Rev.

[75] Hammer ND, Skaar EP (2011). Molecular mechanisms of *Staphylococcus aureus* iron acquisition. Annu Rev Microbiol.

[76] André J-B, Hochberg ME (2005). Virulence evolution in emerging infectious diseases. Evolution.

[77] Hawley DM, Osnas EE, Dobson AP, Hochachka WM, Ley DH, Dhondt AA (2013). Parallel patterns of increased virulence in a recently emerged wildlife pathogen. PLoS Biol.

[78] Altermatt F, Ebert D (2008). Genetic diversity of *Daphnia magna* populations enhances resistance to parasites. Ecol Lett.

[79] Reber A, Castella G, Christe P, Chapuisat M (2008). Experimentally increased group diversity improves disease resistance in an ant species. Ecol Lett.

[80] Hughes WOH, Boomsma JJ (2006). Does genetic diversity hinder parasite evolution in social insect colonies?. J Evol Biol.

[81] Kubinak JL, Cornwall DH, Hasenkrug KJ, Adler FR, Potts WK (2015). Serial infection of diverse host (*Mus*) genotypes rapidly impedes pathogen fitness and virulence. Proc R Soc B.

[82] Finke DL, Snyder WE (2008). Niche Partitioning increases resource exploitation by diverse communities. Science.

[83] Troemel ER, Chu SW, Reinke V, Lee SS, Ausubel FM, Kim DH (2006). p38 MAPK Regulates expression of immune response genes and contributes to longevity in *C. elegans*. PLoS Genet.

[84] Longdon B, Brockhurst MA, Russell CA, Welch JJ, Jiggins FM (2014). The evolution and genetics of virus host shifts. PLOS Pathog.

[85] Longdon B, Hadfield JD, Webster CL, Obbard DJ, Jiggins FM (2011). Host phylogeny determines viral persistence and replication in novel Hosts. PLOS Pathog.

[86] Charleston MA, Robertson DL (2002). Preferential host switching by primate Lentiviruses can account for phylogenetic similarity with the primate phylogeny. Syst Biol.

[87] Streicker DG, Altizer SM, Velasco-Villa A, Rupprecht CE (2012). Variable evolutionary routes to host establishment across repeated rabies virus host shifts among bats. Proc Natl Acad Sci USA.

[88] Salazar-Jaramillo L, Paspati A, van de Zande L, Vermeulen CJ, Schwander T, Wertheim B (2014). Evolution of a cellular immune response in *Drosophila*: a phenotypic and genomic comparative analysis. Genome Biol Evol.

[89] Mollentze N, Biek R, Streicker DG (2014). The role of viral evolution in rabies host shifts and emergence. Curr Opin Virol.

[90] Gervasi SS, Stephens PR, Hua J, Searle CL, Xie GY, Urbina J (2017). Linking ecology and epidemiology to understand predictors of multi-host responses to an emerging pathogen, the amphibian *Chytrid* fungus. PLoS ONE.

[91] Cleaveland S, Laurenson MK, Taylor LH (2001). Diseases of humans and their domestic mammals: pathogen characteristics, host range and the risk of emergence. Philos Trans R Soc B Biol Sci.

[92] Woolhouse MEJ (2001). Population biology of multihost pathogens. Science.

[93] Bonneaud C, Weinert LA, Kuijper B (2019). Understanding the emergence of bacterial pathogens in novel hosts. Philos Trans R Soc B Biol Sci.

[94] Matuszewska M, Murray GGR, Harrison EM, Holmes MA, Weinert LA (2020). The evolutionary genomics of host specificity in *Staphylococcus aureus*. Trends Microbiol.

[95] Spoor LE, McAdam PR, Weinert LA, Rambaut A, Hasman H, Aarestrup FM, et al. Livestock origin for a human pandemic clone of community-associated Methicillin-resistant *Staphylococcus aureus*. mBio. 2013;4. 10.1128/mBio.00356-13. 10.1128/mBio.00356-13 PMC3747577 23943757

[96] Lowder BV, Fitzgerald JR (2010). Human origin for avian pathogenic *Staphylococcus aureus*. Virulence.

[97] Viana D, Comos M, McAdam PR, Ward MJ, Selva L, Guinane CM (2015). A single natural nucleotide mutation alters bacterial pathogen host tropism. Nat Genet.

[98] Monecke S, Gavier-Widén D, Hotzel H, Peters M, Guenther S, Lazaris A (2016). Diversity of *Staphylococcus aureus* isolates in European wildlife. PLOS ONE.

[99] Lowder BV, Guinane CM, Zakour NLB, Weinert LA, Conway-Morris A, Cartwright RA (2009). Recent human-to-poultry host jump, adaptation, and pandemic spread of *Staphylococcus aureus*. Proc Natl Acad Sci USA.

[100] García-Lara J, Needham AJ, Foster SJ (2005). Invertebrates as animal models for *Staphylococcus aureus* pathogenesis: a window into host–pathogen interaction. FEMS Immunol Med Microbiol.

[101] Dieckmann U, Metz JAJ, Sabelis MW. Adaptive dynamics of infectious diseases. In: Pursuit of virulence management. New York: Cambridge University Press; 2005.

