# Supplementary material for: Host genotype and genetic diversity shape the evolution of a novel bacterial infection
Source: ISME J. 2021 Feb 18;15(7):2146–57. doi: 10.1038/s41396-021-00911-3 (PMC8245636; doi:10.1038/s41396-021-00911-3)
Supplement: Supplementary file 1 — Supplemental material [file 41396_2021_911_MOESM1_ESM.pdf]

Supplementary Information for

Host genotype and genetic diversity shape the evolution of a novel bacterial infection

Alice K.E. Ekroth*, Michael Gerth, Emily J. Stevens, Suzanne A. Ford, and Kayla C. King*

Corresponding authors: alice.ekroth1@gmail.com, kayla.king@zoo.ox.ac.uk

This file contains:

Materials and Methods

Figures S1-S4

Tables S1-S3

Materials and Methods

GO term enrichment analysis

We conducted gene pairwise comparisons across selected worm isolate genomes. We selected comparisons based on whether pathogen populations selected within those host genotypes evolved different or similar virulence levels (data shown in Fig.1). We then created a list of any fixed variant within a protein coding gene per nematode isolate. A pairwise comparison was then conducted between nematode isolate pairs where we discarded any shared loci, thus creating a list of unique variant loci for each worm genotype pair. We hypothesized that these unique loci could be drivers of the observed pathogen traits emerging during evolution within those nematode genotypes. We randomly chose combinations from our list of unique variants and performed a gene ontology (GO) enrichment analysis using g:Profiler (https://biit.cs.ut.ee/gprofiler/gost). The resulting GO terms were then listed in Table S2.

Gene determination of SNP data

For each SNP, we confirmed the corresponding gene, and then the putative product and gene function through a literature search (Supp. Table 3). Gene products were placed under the following function categories; adherence, adherence and metabolism, virulence and adherence, metabolism, metabolism and stress, stress, virulence and stress, virulence, virulence and metabolism, siderophore and metabolism, siderophore, and unknown. Within-population SNP frequencies were calculated by adding up frequencies and dividing by the number of occurrences of a particular gene product. An average of total gene products of a particular function was then compared between host population treatments. We also combined all virulence related functions to evaluate differences between host population treatments. We only statistically compared functions where > 1 SNP was found. As these data were not normally distributed, we used Wilcoxon rank sum tests to compare average snp frequencies of functions between monoculture and polyculture treatments.

Siderophore production

Ancestral and evolved populations of *S. aureus* were grown overnight in 5 mL TSB at 220rpm at 30°C. Populations were then standardised by OD630, and subsequently filtered through 22 µm filters to remove bacterial cells. Instructions supplied by the SideroTech (EmergenBio) kit were followed by taking 100 µL of each sample to prepared 100 µL reaction mixture and left at r.t.p for 10 min before OD630 measures were taken. Siderophore concentrations are estimated by measuring the changes in OD630 as the result of ferric iron transfer from the reagent (composed of a dye, iron, and a detergent) to siderophore present. Standards supplied by the kit were used to create a reference curve from which the relationship between desferoxamine siderophore and OD630 could be measured and siderophores present in the samples could be determined.

To determine if siderophore production related to pathogen-induced host mortality, we conducted Pearson's product-moment correlation and Spearman's rank correlation tests

between host mortality and siderophore levels for pathogen populations in polycultures and monocultures, respectively.

Phylogenetic tree calculation

To determine whether phylogenetic clustering was observed in our pathogen populations evolved within host monocultures and polycultures, we created phylogenetic trees using the Euclidean distance data described in the main text *methods*. A phylogenetic tree (Fig. S5) was calculated by hierarchical clustering of Euclidean distances. Due to unequal variances, a Welch two sample t-test was used to determine differences between monoculture and polyculture treatment.

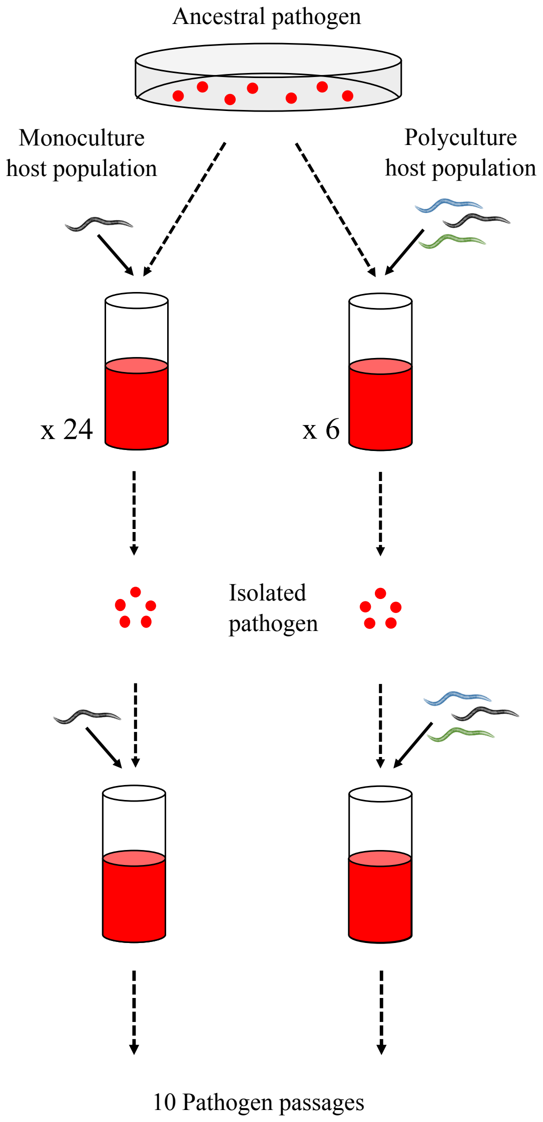

Figure S1: Experimental evolution of pathogens in host genotypes and population types. *Staphylococcus aureus* was passaged in monocultures of 24 nematode host genotypes and polycultures (six replicate populations, each a pool of 24 nematode isolates) across ten host generations from a single ancestral clone. Pathogens were presented to new hosts populations from stock culture at each passage. Nematodes were exposed to pathogens for 24h, after which 10% of all exposed hosts in the replicate were isolated, crushed, and pathogens was used to re-infect new nematodes. To control for lab adaptation on genomic evolution, the pathogen was also passaged *in vitro* in six replicate no-host controls.

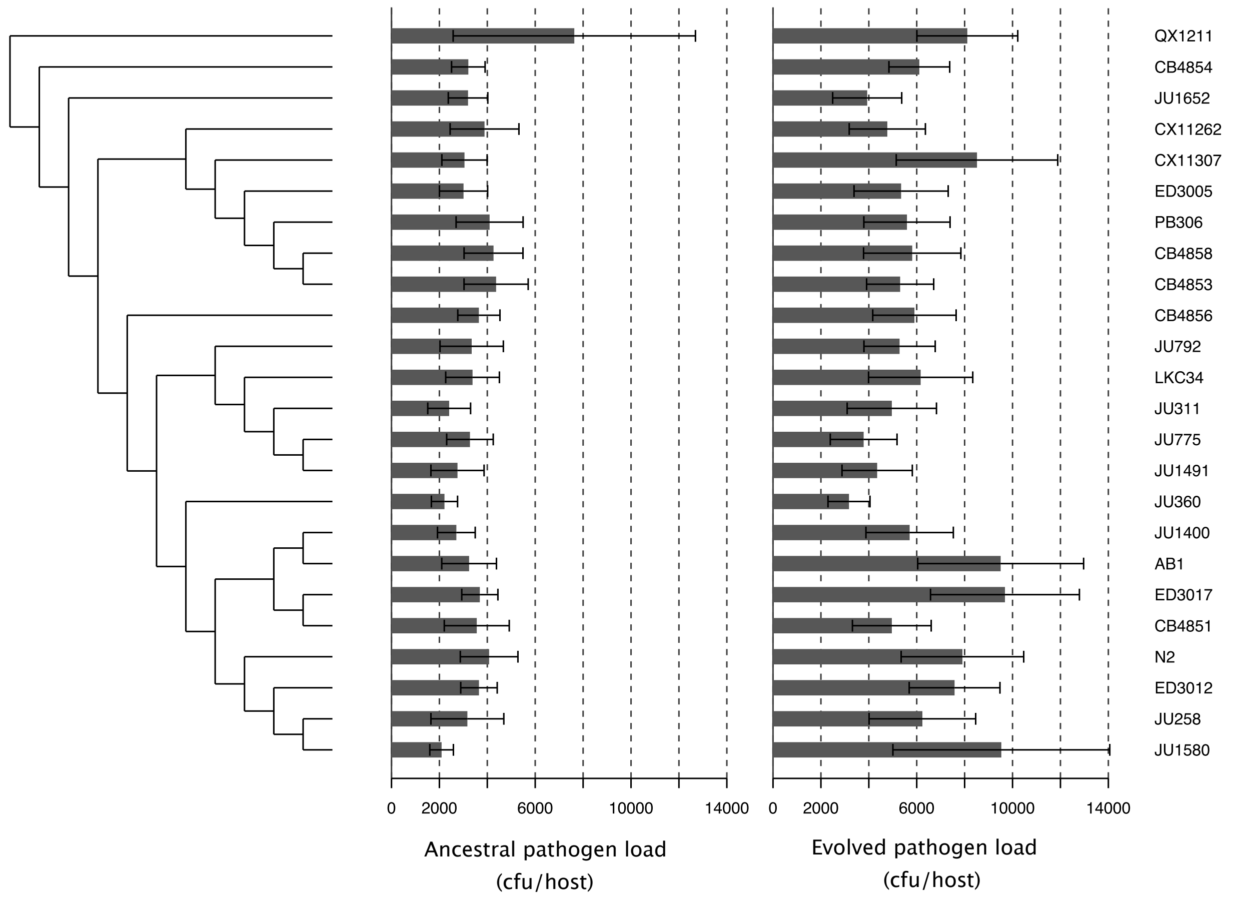

Figure S2: Phylogeny of *C. elegans* strains mapped against pathogen infection load. Monocultures of 24 *C. elegans* strains were infected with ancestral or evolved *S. aureus* for 24h, whereby infection load (mean ± se cfu/host) was measured. Assays of pathogen load consisted of five replicates. The tree is rooted by the most diverse host strain (QX1121), see Andersen et al. 2012 for a full description. Statistical analysis of variation across host genotypes can be found in Table 1 of main text.

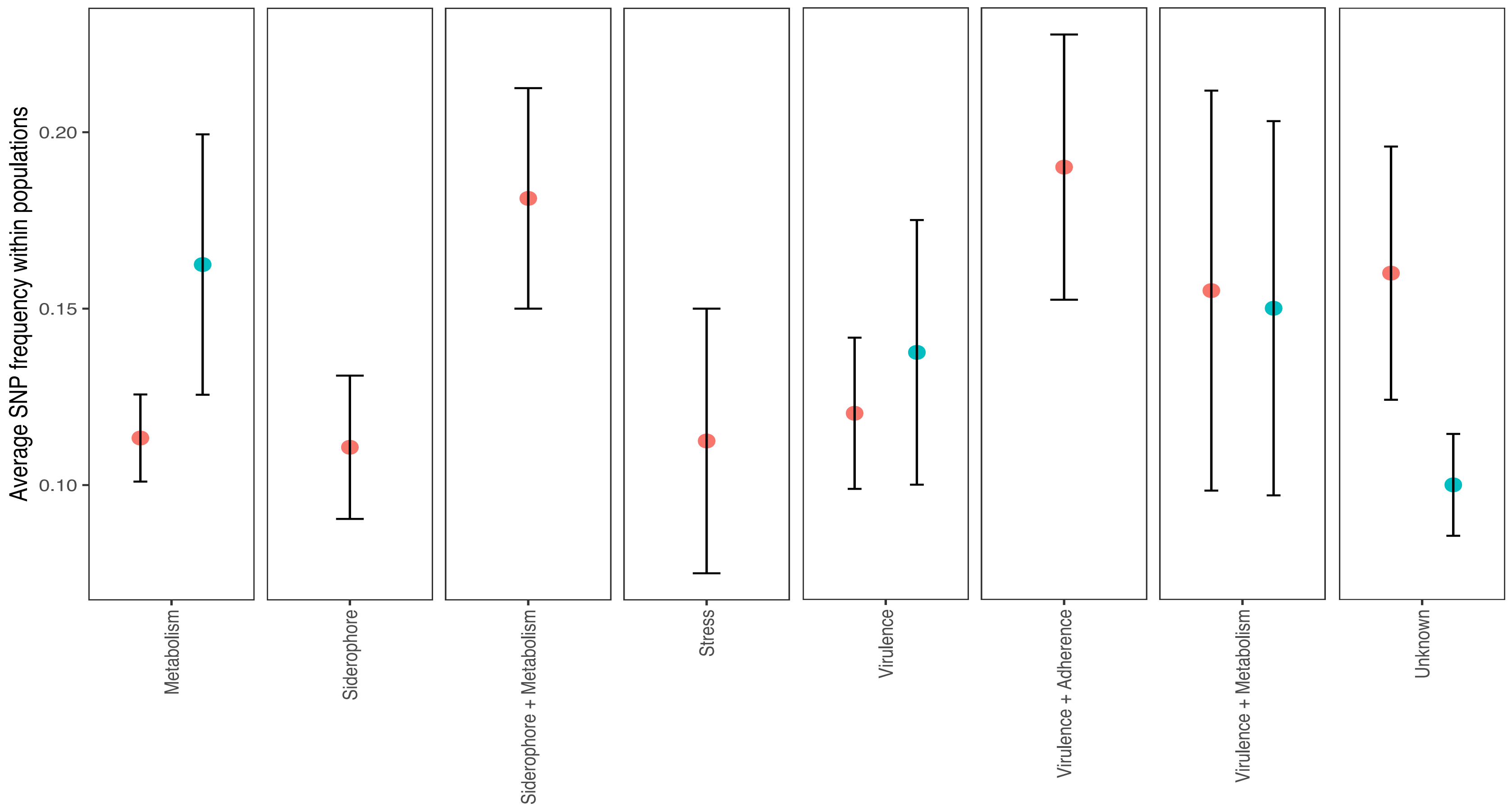

Figure S3: Within-population SNP frequencies in experimentally evolved pathogen populations. SNP frequencies (mean ± SE) are shown for pathogens evolved in host monocultures (red) and polycultures (blue). Wilcoxon rank sum tests did not reveal differences in SNP frequencies between pathogens evolved in host monocultures and polycultures for functions related to: Metabolism ($p = 0.2796$), Virulence ($p = 0.7639$), Virulence + Metabolism ($p = 1$) or Unknown ($p = 0.3653$). Where SNPs were only found in monoculture pathogens, Wilcoxon rank sum test of functions related to Siderophores ($p = 0.0213$) was significant from zero, but not for Siderophore + Metabolism ($p = 0.5$), Stress ($p = 0.5$), or Virulence + Adherence ($p = 0.0625$)

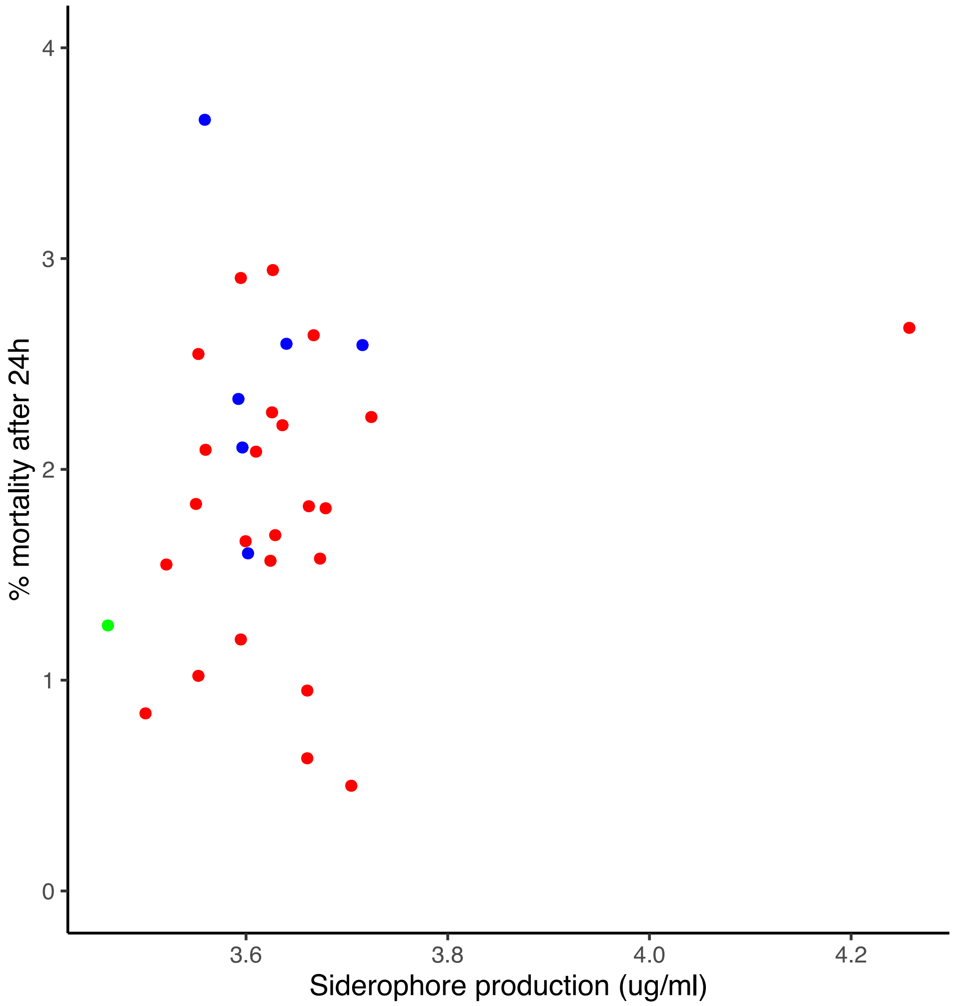

Figure S4: Pathogen virulence and siderophore production. Pathogen-induced host mortality (% dead nematodes after 24h pathogen exposure) plotted against siderophore production (µg/mL) for ancestral (green), monoculture-evolved (red), and polyculture-evolved (blue) *S. aureus.* Siderophore production did not differ between pathogens evolved within between host population types (ANOVA, X^2 = 2.899, d.f. = 2, p = 0.235). Siderophore production was also not correlated with host killing ability by pathogens evolved in monocultures (Spearman's rank correlation, rho = 0.12, p = 0.57) or polycultures (Pearson's product-moment correlation, t = -033, cor = -0.16, p = 0.76).

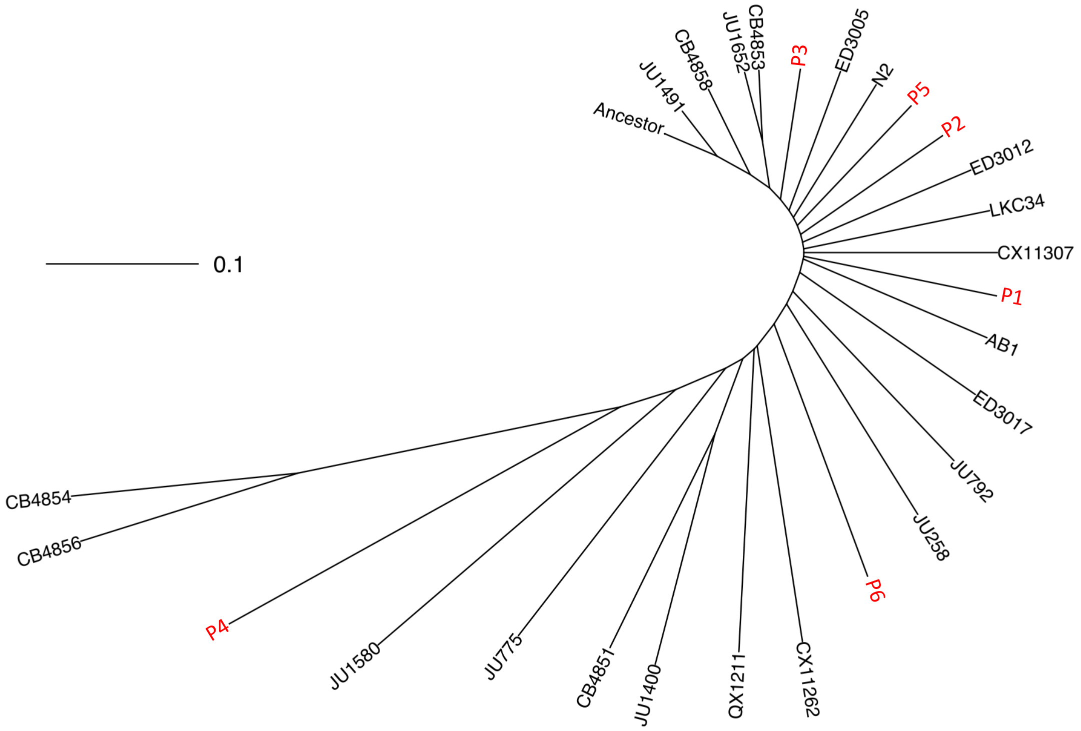

Figure S5: Euclidean genetic distance of evolved pathogen populations in host genotypes in monoculture and polyculture. Phylogenetic tree measuring Euclidean distance of evolved *S. aureus* populations from the ancestor after 10 passages in host monocultures (names of wild isolates shown) and polycultures (replicates P1-P6). Euclidean distances are measured from pairwise comparisons of evolved pathogen populations using SNP frequencies. Pathogens populations evolved within host genotypes JU311, JU363, PB306 did not differ significantly from the ancestor. We found no significant difference between Euclidean distances of monoculture and polyculture populations (Welch two sample t-test, d.f. = 7.56, t = 0.355, p = 0.736).

Table S1: *C. elegans* strains used in the study. Information obtained from *Caenorhabditis elegans* Natural Diversity Resource (https://www.elegansvariation.org).

Strain	City, Nation, or State	Isolation Date	Sampled by
**AB1**	Adelaide, Australia	1983	D. Riddle and A. Bird
**CB4851**	Bergerac, France	Pre-1949	V. Nigon
**CB4853**	Altadena, USA	1974	C.D. Johnson
**CB4854**	Altadena, USA	1974	C.D. Johnson
**CB4856**	Oahu, Hawaii	1972	L. Hollen
**CB4857**	Claremont, USA	1972	E.M. Hedgecock
**CB4858**	Pasadena, USA	1973	E.M. Hedgecock
**CX11262**	Los Angeles, USA	2003	A. Sivasundar
**CX11307**	Los Angeles, USA	2003	A. Sivasundar
**ED3005**	Edinburgh, United Kingdom	2004	A. Cutter
**ED3012**	Edinburgh, United Kingdom	2004	A. Cutter
**ED3017**	Edinburgh, United Kingdom	2004	A. Cutter
**JU258**	Ribeiro, Madeira	2001	M-A. Félix
**JU311**	Merlet, France	2002	M-A. Félix
**JU363**	Franconville, France	2002	C. Pieau
**JU775**	Lisbon, Portugal	2005	M-A. Félix
**JU792**	Fréchendets, France	2005	M-A. Félix
**JU1400**	Sevilla, Spain	2008	M-A. Félix
**JU1491**	Le Blanc, France	2008	M-A. Félix
**JU1580**	Orsay, France	2008	M-A. Félix
**JU1652**	Montevideo, Uruguay	2009	NA
**LKC34**	Unknown city, Madagascar	2005	V. Stowell
**N2**	Bristol, UK	1951	L.N. Staniland and W. Nicholas
**PB306**	NA	1998	S. Baird
**QX1211**	San Francisco, USA	2007	M. Rockman

Table S2: GO term results of *C. elegans* genotype comparisons. Shown are the molecular function comparisons of host genotypes varying in the virulence outcomes from their sympatric pathogens. Highlighted in red are terms that putatively relate to metal ion binding functions in nematodes.

Different virulence	Similar virulence
**JU775 vs. JU1580**	**QX1211 vs. ED3012**
**cation binding**<br>**transition metal ion binding**<br>**zinc ion binding**<br>**metal ion binding**	transferase activity, transferring glycosyl groups<br>catalytic activity, acting on RNA<br>transferase activity, transferring hexosyl groups<br>transferase activity<br>catalytic activity<br>fucosyltransferase activity<br>UDP-glycosyltransferase activity
**JU1400 vs CB4854**	**JU1652 vs JU311**
G protein-coupled olfactory receptor activity<br>G protein-coupled receptor activity<br>olfactory receptor activity<br>acetylgalactosaminyltransferase activity<br>DNA-binding transcription factor activity<br>G protein-coupled peptide receptor activity<br>**transition metal ion binding**<br>sequence-specific DNA binding<br>peptide receptor activity<br>transmembrane signalling receptor activity<br>**zinc ion binding**	ligase activity, forming carbon-nitrogen bonds
**CB4851 vs CB4858**	**CB4856 vs AB1**
**ion transmembrane transporter activity**<br>**ion channel activity**<br>inorganic molecular entity transmembrane transporter activity<br>**protein binding**<br>binding<br>**cation channel activity**<br>small GTPase binding<br>gated channel activity<br>channel activity<br>passive transmembrane transporter activity<br>**ion gated channel activity**<br>Ras GTPase binding	peptide receptor activity
**ED3017 vs JU1652**	**CB4854 VS N2**

steroid hydroxylase activity<br>G protein-coupled peptide receptor activity<br>oxidoreductase activity, acting on paired donors, with incorporation or reduction of molecular oxygen<br>oxidoreductase activity, acting on paired donors, with incorporation or reduction of molecular oxygen, reduced flavin or flavoprotein as one donor, and incorporation of one atom of oxygen<br>peptide receptor activity<br>**transition metal ion binding**<br>DNA-binding transcription factor activity<br>**iron ion binding**<br>sequence-specific DNA binding<br>transcription regulator activity<br>monooxygenase activity	anion binding<br>adenyl ribonucleotide binding<br>adenyl nucleotide binding<br>purine ribonucleotide binding<br>purine nucleotide binding<br>ATP binding<br>nucleoside phosphate binding<br>nucleoside phosphate binding<br>nucleotide binding<br>transcription coregulator activity<br>purine ribonucleoside triphosphate binding<br>ribonucleotide binding<br>carbohydrate derivative binding<br>small molecule binding<br>protein binding<br>**ion transmembrane transporter activity**<br>**ion gated channel activity**<br>drug binding<br>gated channel activity<br>binding<br>voltage-gated channel activity<br>**voltage-gated ion channel activity**<br>inorganic molecular entity transmembrane transporter activity<br>catalytic activity, acting on a protein<br>**ion channel activity**
**CX11262 vs N2**	**JU1652 vs JU792**
phosphorus-oxygen lyase activity<br>**voltage-gated ion channel activity**<br>voltage-gated channel activity<br>cyclase activity<br>guanylate cyclase activity<br>binding<br>**protein binding**<br>**voltage-gated cation channel activity**<br>potassium ion transmembrane transporter activity<br>gated channel activity	DNA-binding transcription factor activity<br>transcription regulator activity
**JU775 vs. JU1491**	**JU258 vs. JU1580**
G protein-coupled receptor activity<br>**transition metal ion binding**<br>signalling receptor activity<br>transmembrane signalling receptor activity<br>molecular transducer activity<br>**zinc ion binding**<br>oxidoreductase activity<br>G protein-coupled olfactory receptor activity	UDP-glycosyltransferase activity<br>transferase activity, transferring glycosyl groups<br>transferase activity, transferring hexosyl groups

steroid hydroxylase activity<br>sequence-specific DNA binding<br>DNA-binding transcription factor activity<br>olfactory receptor activity<br>aspartic-type endopeptidase activity<br>aspartic-type peptidase activity<br>G protein-coupled peptide receptor activity<br>**metal ion binding**<br>**cation binding**<br>transcription regulator activity<br>peptide receptor activity	
**ED3017 vs N2**	**JU792 vs LKC34**
gated channel activity<br>sequence-specific DNA binding<br>**ion gated channel activity**<br>voltage-gated channel activity<br>**voltage-gated ion channel activity**<br>DNA-binding transcription factor activity<br>**ion channel activity**<br>transcription regulator activity<br>voltage-gated cation channel activity<br>voltage-gated potassium channel activity<br>potassium channel activity<br>**ion transmembrane transporter activity**	carbohydrate binding
**CB4858 vs ED3005**	**CX11307 vs ED3005**
G protein-coupled olfactory receptor activity<br>olfactory receptor activity<br>G protein-coupled receptor activity	G protein-coupled receptor activity<br>olfactory receptor activity<br>signalling receptor activity
**ED3012 VS JU258**	**CB4856 VS CB4853**
sequence-specific DNA binding	DNA-binding transcription factor activity<br>sequence-specific DNA binding<br>G protein-coupled receptor activity<br>peptide receptor activity<br>G protein-coupled peptide receptor activity<br>**transition metal ion binding**<br>**transferase activity, transferring glycosyl groups**<br>transcription regulator activity<br>signalling receptor activity<br>UDP-glycosyltransferase activity<br>molecular transducer activity<br>oxidoreductase activity<br>**zinc ion binding**<br>steroid hydroxylase activity

	transmembrane signalling receptor activity<br>transferase activity, transferring hexosyl groups<br>**metal ion binding**<br>**cation binding**<br>oxidoreductase activity<br>carbohydrate binding
**QX1211 vs CB4854**	**JU1491 VS JU363**
transferase activity, transferring glycosyl groups<br>UDP-glycosyltransferase activity<br>oxidoreductase activity<br>**transition metal ion binding**<br>steroid hydroxylase activity<br>transferase activity, transferring hexosyl groups<br>sequence-specific DNA binding<br>**zinc ion binding**<br>G protein-coupled receptor activity<br>DNA-binding transcription factor activity<br>G protein-coupled olfactory receptor activity<br>glucuronosyltransferase activity	acetylgalactosaminyltransferase activity<br>**ion binding**

Table S3: SNP data results with gene function allocation of gene products in pathogens evolved in host monocultures and polycultures.

Position	Reference	SNP	Gene tag	Gene product	Host population	Function
534776	T	G	SAS_RS02555	16S ribosomal RNA	Mono	**Metabolism**[3]
1434373	T	A	SAS_RS07090	2,3,4,5-tetrahydropyridine-2,6-dicarboxylate N-acetyltransferase	Mono & Poly	**Metabolism**[4]
2116677	G	A	SAS_RS10675	23S ribosomal RNA	Poly	**Metabolism**[5]
2290705	G	T	SAS_RS11560	30S ribosomal protein S5	Mono	**Metabolism**[6]
508473	T	G	SAS_RS02380	4-diphosphocytidyl-2C-methyl-D-erythritol kinase	Mono	**Metabolism**[7]
2734062	G	A	SAS_RS13830	accessory Sec system protein Asp1	Poly	**Virulence**[8]
252031	A	G	SAS_RS01080-SAS_RS01085	acyl CoA:acetate/3-ketoacid CoA transferase - PrsW family intramembrane metalloprotease	Poly	**Virulence + Adherence**[9]
1693527	C	T	SAS_RS08335	adenine phosphoribosyltransferase	Mono	**Metabolism**[10]
1362660	G	A	SAS_RS06750-SAS_RS06755	amino acid permease - catalase	Mono	**Siderophore**[11]
1595090	C	G	SAS_RS07805	aminopeptidase P family protein	Mono	**Metabolism**[12]
384882	G	A	SAS_RS01735	aminotransferase class I/II-fold pyridoxal phosphate-dependent enzyme	Poly	**Unknown**
1326110	C	A	SAS_RS06535	aquaporin family protein	Mono	**Metabolism**[13]
1431962	C	T	SAS_RS07075	aspartate-semialdehyde dehydrogenase	Poly	**Virulence + Metabolism**[14]
1548501	T	C	SAS_RS07560	ATP-dependent DNA helicase RecQ - reduces nematode killing	Mono	**Virulence**[15]
1900576	A	T	SAS_RS09310-SAS_RS09315	beta-channel forming cytolysin	Mono	**Virulence**[16]
1059975	TAGCTTTAGGTGCTGATGTAGTTGC	T	SAS_RS05230	bifunctional autolysin	Mono	**Adherence + Metabolism**[17]
2703970	T	C	SAS_RS13725	carbamate kinase 2	Mono & Poly	**Metabolism**[18]
304315	G	T	SAS_RS01330-SAS_RS01335	CHAP domain-containing protein - WXG100 family type	Mono	**Siderophore**[19]

				VII secretion effector EsxA		
1034531	A	ATTAT	SAS_RS05095	CPBP family intramembrane metalloprotease	Mono	**Metabolism**[20]
2317639	A	T	SAS_RS11750	cyclic pyranopterin monophosphate synthase	Mono	**Metabolism**[21]
2527945	G	T	SAS_RS12845-SAS_RS12850	D-histidine (S)-2-aminobutanoyltransferase CntL - histidine racemase CntK	Mono	**Siderophore**[22]
2195585	ATTCGC	A	SAS_RS11090-SAS_RS11095	deoxyribose-phosphate aldolase - purine-nucleoside phosphorylase	Mono	**Metabolism**[23]
1322701	AGCG	A	SAS_RS06525	DNA mismatch repair protein MutL	Mono	**Metabolism + Stress**[24]
702734	G	A	SAS_RS03350	DNA-binding response regulator	Poly	**Metabolism**[25]
458723	A	G	SAS_RS02130	DUF2309 family protein	Poly	**Unknown**
325676	C	A	SAS_RS01450	DUF4064 domain-containing protein	Mono	**Unknown**
2308383	G	A	SAS_RS11705	efflux RND transporter permease subunit	Mono	**Siderophore**[11]
1083589	A	C	SAS_RS05345	energy-coupling factor transporter transmembrane protein EcfT	Mono	**Siderophore**[26]
1658833	C	A	SAS_RS08165-SAS_RS08170	enterotoxin - Fic family protein	Mono	**Virulence**[27]
1263454	GAAATA	G	SAS_RS06275	FemA/FemB family glycyltransferase FmhC	Mono	**Metabolism**[28]
1163190	C	T	SAS_RS05760-SAS_RS05765	fibrinogen-binding protein	Mono	**Virulence + Adherence**[27]
326572	C	G	SAS_RS01455	formate/nitrite transporter	Poly	**Metabolism**[29]
1332856	G	T	SAS_RS06570	GTPase HflX	Mono	**Metabolism**[30]
1495257	C	T	SAS_RS07310	hyperosmolarity resistance protein Ebh	Mono	**Virulence + Metabolism**[31]
1116202	G	T	SAS_RS05520	hypothetical protein	Mono & Poly	**Unknown**
524447	A	T	SAS_RS02465-SAS_RS02470	hypoxanthine phosphoribosyltransferase - ATP-dependent metallopeptidase FtsH/Yme1/Tma family protein	Mono	**Unknown**
1682025	T	A	SAS_RS08275	LLM class flavin-dependent oxidoreductase	Mono	**Stress**[32]

1578429	C	A	SAS_RS07715	M20/M25/M40 family metallo-hydrolase	Mono	**Stress**[33]
2007983	C	T	SAS_RS10010	manganese-dependent inorganic pyrophosphatase	Mono	**Virulence + Metabolism**[34]
730082	C	A	SAS_RS03495-SAS_RS03500	MarR family transcriptional regulator - GTP-binding protein	Mono	**Virulence**[35]
2220609	G	A	SAS_RS11185	methicillin resistance protein FmtB	Poly	**Virulence + Metabolism**[36]
862171	T	C	SAS_RS04175	methionine import ATP-binding protein MetN 2	Poly	**Adherence + Metabolism**[37]
2420676	C	T	SAS_RS12285	MFS transporter	Mono	**Siderophore + Metabolism**[11]
601843	T	C	SAS_RS02815-SAS_RS02820	MSCRAMM family adhesin SdrD - MSCRAMM family adhesin SdrE	Mono	**Virulence + Adherence**[38]
341627	C	T	SAS_RS01525	N-acetylmannosamine-6-phosphate 2-epimerase	Mono	**Adherence**[39]
957468	G	A	SAS_RS04635	oligoendopeptidase F	Mono	**Virulence + Stress**[40]
1188175	C	A	SAS_RS05930	penicillin-binding protein	Mono	**Virulence + Metabolism**[41]
2027051	C	A	SAS_RS10135-SAS_RS10140	peptidoglycan hydrolase	Mono	**Virulence + Metabolism**[42]
197793	A	G	SAS_RS00855	permease	Mono	**Siderophore**[11]
1224242	C	A	SAS_RS06085-SAS_RS06090	primosomal protein N'	Mono	**Metabolism**[43]
440881	T	A	SAS_RS02050	restriction endonuclease subunit S	Poly	**Metabolism**[44]
1098392	G	A	SAS_RS05420	ribonuclease J 1	Poly	**Virulence + Metabolism**[45]
800534	C	CA	SAS_RS03855-SAS_RS03860	ribosome-associated translation inhibitor RaiA - preprotein translocase subunit SecA	Mono	**Siderophore**[46]
2743155	C	A	SAS_RS13840	serine-rich repeat glycoprotein adhesin SasA	Mono	**Virulence + Adherence**[47]
1986449	G	A	SAS_RS09915	sodium:proline symporter	Mono	**Siderophore + Metabolism**[11]
2066931	TGCCAA	T	SAS_RS10420-SAS_RS10425	staphylococcal enterotoxin type K - site-specific integrase	Mono	**Virulence**[48]
99839	G	T	SAS_RS00420-SAS_RS14315	staphylococcal protei n A	Mono	**Virulence + Adherence**[49]

445553	G	A	SAS_RS02070	tandem-type lipoprotein	Mono & Poly	**Virulence**[50]
848777	C	A	SAS_RS04065	thermonuclease - biofilm formation	Mono	**Virulence + Adherence**[51]
1368440	A	G	SAS_RS06785-SAS_RS06790	transcriptional repressor LexA - hypothetical protein	Poly	**Metabolism**[52]
635932	G	A	SAS_RS02985	transposase	Mono	**Unknown**
1408853	G	T	SAS_RS06960	tryptophan synthase subunit alpha	Mono	**Virulence + Metabolism**[53]
1959839	G	A	SAS_RS09760	type 1 glutamine amidotransferase	Mono	**Metabolism**[54]
1791153	TA	T	SAS_RS08805-SAS_RS08810	tyrosine--tRNA ligase - penicillin-binding protein	Mono	**Virulence**[55]
1545839	C	T	SAS_RS07545	YpdA family putative bacillithiol disulfide reductase	Poly	**Virulence + Metabolism**[56]
2402539	G	A	SAS_RS12205	zinc ribbon domain-containing protein	Mono	**Unknown**

References

1. Kawecki, T. J. & Ebert, D. Conceptual issues in local adaptation. *Ecology Letters* **7**, 1225–1241 (2004).
2. Morley, D., Broniewski, J. M., Westra, E. R., Buckling, A. & Houte, S. van. Host diversity limits the evolution of parasite local adaptation. *Molecular Ecology* **26**, 1756–1763 (2017).
3. Brimacombe, R. The emerging three-dimensional structure and function of 16S ribosomal RNA. *Biochemistry* **27**, 4207–4214 (1988).
4. Whiteley, A. T. *et al.* c-di-AMP modulates Listeria monocytogenes central metabolism to regulate growth, antibiotic resistance and osmoregulation. *Molecular Microbiology* **104**, 212–233 (2017).
5. Bokov, K. & Steinberg, S. V. A hierarchical model for evolution of 23S ribosomal RNA. *Nature* **457**, 977–980 (2009).
6. Ramakrishnan, V. & White, S. W. The structure of ribosomal protein S5 reveals sites of interaction with 165 rRNA. *Nature* **358**, 768–771 (1992).
7. Miallau, L. *et al.* Biosynthesis of isoprenoids: Crystal structure of 4-diphosphocytidyl-2C-methyl-d-erythritol kinase. *PNAS* **100**, 9173–9178 (2003).
8. Siboo, I. R., Chaffin, D. O., Rubens, C. E. & Sullam, P. M. Characterization of the Accessory Sec System of Staphylococcus aureus. *Journal of Bacteriology* **190**, 6188–6196 (2008).
9. Ho, T. D. & Ellermeier, C. D. PrsW Is Required for Colonization, Resistance to Antimicrobial Peptides, and Expression of Extracytoplasmic Function σ Factors in Clostridium difficile. *Infection and Immunity* **79**, 3229–3238 (2011).
10. Craig, S. P. & Eakin, A. E. Purine Phosphoribosyltransferases. *J. Biol. Chem.* **275**, 20231–20234 (2000).
11. Miethke, M. & Marahiel, M. A. Siderophore-Based Iron Acquisition and Pathogen Control. *Microbiology and Molecular Biology Reviews* **71**, 413–451 (2007).
12. Singh, A. K., Singh, R., Tomar, D., Pandya, C. D. & Singh, R. The leucine aminopeptidase of Staphylococcus aureus is secreted and contributes to biofilm formation. *International Journal of Infectious Diseases* **16**, e375–e381 (2012).
13. Takata, K., Matsuzaki, T. & Tajika, Y. Aquaporins: water channel proteins of the cell membrane. *Progress in Histochemistry and Cytochemistry* **39**, 1–83 (2004).
14. Luniwal, A., Wang, L., Pavlovsky, A., Erhardt, P. W. & Viola, R. E. Molecular docking and enzymatic evaluation to identify selective inhibitors of aspartate semialdehyde dehydrogenase. *Bioorganic & Medicinal Chemistry* **20**, 2950–2956 (2012).
15. Bae, T. *et al.* Staphylococcus aureus virulence genes identified by bursa aurealis mutagenesis and nematode killing. *PNAS* **101**, 12312–12317 (2004).
16. Surmann, K. *et al.* Analysis of Staphylococcus aureus proteins secreted inside infected human epithelial cells. *International Journal of Medical Microbiology* **308**, 664–674 (2018).
17. Götz, F., Heilmann, C. & Stehle, T. Functional and structural analysis of the major amidase (Atl) in Staphylococcus. *International Journal of Medical Microbiology* **304**, 156–163 (2014).
18. Sendi, P. & Proctor, R. A. Staphylococcus aureus as an intracellular pathogen: the role of small colony variants. *Trends in Microbiology* **17**, 54–58 (2009).
19. Houben, E. N. G., Korotkov, K. V. & Bitter, W. Take five — Type VII secretion systems of Mycobacteria. *Biochimica et Biophysica Acta (BBA) - Molecular Cell Research* **1843**, 1707–1716 (2014).

20. Schaeffer, R. D. *et al.* ECOD: identification of distant homology among multidomain and transmembrane domain proteins. *BMC Molecular and Cell Biology* **20**, 1–11 (2019).
21. Leimkühler, S., Wuebbens, M. M. & Rajagopalan, K. V. The history of the discovery of the molybdenum cofactor and novel aspects of its biosynthesis in bacteria. *Coordination Chemistry Reviews* **255**, 1129–1144 (2011).
22. Ghssein, G. *et al.* Biosynthesis of a broad-spectrum nicotianamine-like metallophore in Staphylococcus aureus. *Science* **352**, 1105–1109 (2016).
23. Yadav, P. K., Singh, G., Singh, S., Gautam, B. & Saad, E. I. Potential therapeutic drug target identification in Community Acquired-Methicillin Resistant Staphylococcus aureus (CA-MRSA) using computational analysis. *Bioinformation* **8**, 664–672 (2012).
24. Prunier, A.-L. & Leclercq, R. Role of mutS and mutL Genes in Hypermutability and Recombination in Staphylococcus aureus. *Journal of Bacteriology* **187**, 3455–3464 (2005).
25. Galperin, M. Y. Diversity of structure and function of response regulator output domains. *Current Opinion in Microbiology* **13**, 150–159 (2010).
26. Prava, J., G, P. & Pan, A. Functional assignment for essential hypothetical proteins of Staphylococcus aureus N315. *International Journal of Biological Macromolecules* **108**, 765–774 (2018).
27. Bhatia, A. & Zahoor, S. Staphylococcus Aureus Enterotoxins: A Review. *Journal of Clinical and Diagnostic research* **3**, 188–197 (2007).
28. Tschierske, M. *et al.* Identification of three additional femAB-like open reading frames in Staphylococcus aureus. *FEMS Microbiol Lett* **171**, 97–102 (1999).
29. Lü, W. *et al.* The formate/nitrite transporter family of anion channels. *Biological Chemistry* **394**, 715–727 (2013).
30. Zhang, Y. *et al.* HflX is a ribosome-splitting factor rescuing stalled ribosomes under stress conditions. *Nature Structural & Molecular Biology* **22**, 906–913 (2015).
31. Cheng, A. G., Missiakas, D. & Schneewind, O. The Giant Protein Ebh Is a Determinant of Staphylococcus aureus Cell Size and Complement Resistance. *Journal of Bacteriology* **196**, 971–981 (2014).
32. Li, S.-W., Liu, M.-Y. & Yang, R.-Q. Comparative Genome Characterization of a Petroleum-Degrading *Bacillus subtilis* Strain DM2. *International Journal of Genomics* **2019**, 1–16 (2019).
33. Marizcurrena, J. J. *et al.* Validating biochemical features at the genome level in the Antarctic bacterium Hymenobacter sp. strain UV11. *FEMS Microbiol Lett* **366**, 1–10 (2019).
34. Gajadeera, C. S., Zhang, X., Wei, Y. & Tsodikov, O. V. Structure of inorganic pyrophosphatase from Staphylococcus aureus reveals conformational flexibility of the active site. *Journal of Structural Biology* **189**, 81–86 (2015).
35. Grove, A. MarR family transcription factors. *Current Biology* **23**, R142–R143 (2013).
36. Bai, J. *et al.* The role of ArlRS in regulating oxacillin susceptibility in methicillin-resistant Staphylococcus aureus indicates it is a potential target for antimicrobial resistance breakers. *Emerging Microbes & Infections* **8**, 503–515 (2019).
37. Krismer, B. *et al.* Nutrient Limitation Governs Staphylococcus aureus Metabolism and Niche Adaptation in the Human Nose. *PLOS Pathogens* **10**, e1003862 (2014).
38. Foster, T. J. & Höök, M. Surface protein adhesins of Staphylococcus aureus. *Trends in Microbiology* **6**, 484–488 (1998).

39. North, R. A. *et al.* Cloning, expression, purification, crystallization and preliminary X-ray diffraction analysis of N-acetylmannosamine-6-phosphate 2-epimerase from methicillin-resistant Staphylococcus aureus. *Acta Crystallographica Section F* **70**, 650–655 (2014).
40. Smith, A., Rowan, R., McCann, M. & Kavanagh, K. Exposure of Staphylococcus aureus to silver(I) induces a short term protective response. *BioMetals* **25**, 611–616 (2012).
41. Georgopapadakou, N. H., Dix, B. A. & Mauriz, Y. R. Possible physiological functions of penicillin-binding proteins in Staphylococcus aureus. *Antimicrobial Agents and Chemotherapy* **29**, 333–336 (1986).
42. Vollmer, W., Joris, B., Charlier, P. & Foster, S. Bacterial peptidoglycan (murein) hydrolases. *FEMS Microbiol Rev* **32**, 259–286 (2008).
43. Lasken, R. S. & Kornberg, A. The primosomal protein n' of Escherichia coli is a DNA helicase. *J. Biol. Chem.* **263**, 5512–5518 (1988).
44. Sadykov, M. R. Restriction–Modification Systems as a Barrier for Genetic Manipulation of Staphylococcus aureus. in *The Genetic Manipulation of Staphylococci: Methods and Protocols* (ed. Bose, J. L.) 9–23 (Springer, 2016). doi:10.1007/7651_2014_180.
45. Roux, C. M., DeMuth, J. P. & Dunman, P. M. Characterization of Components of the Staphylococcus aureus mRNA Degradosome Holoenzyme-Like Complex. *Journal of Bacteriology* **193**, 5520–5526 (2011).
46. Fiester, S. E. *et al.* Role of the Carboxy Terminus of SecA in Iron Acquisition, Protein Translocation, and Virulence of the Bacterial Pathogen Acinetobacter baumannii. *Infection and Immunity* **83**, 1354–1365 (2015).
47. Kukita, K. *et al.* Staphylococcus aureus SasA Is Responsible for Binding to the Salivary Agglutinin gp340, Derived from Human Saliva. *Infection and Immunity* **81**, 1870–1879 (2013).
48. Orwin, P. M., Leung, D. Y. M., Donahue, H. L., Novick, R. P. & Schlievert, P. M. Biochemical and Biological Properties of Staphylococcal Enterotoxin K. *Infect Immun* **69**, 360–366 (2001).
49. Hartleib, J. *et al.* Protein A is the von Willebrand factor binding protein onStaphylococcus aureus. *Blood* **96**, 2149–2156 (2000).
50. Muñoz-Planillo, R., Franchi, L., Miller, L. S. & Núñez, G. A Critical Role for Hemolysins and Bacterial Lipoproteins in Staphylococcus aureus-Induced Activation of the Nlrp3 Inflammasome. *The Journal of Immunology* **183**, 3942–3948 (2009).
51. Mann, E. E. *et al.* Modulation of eDNA Release and Degradation Affects Staphylococcus aureus Biofilm Maturation. *PLOS ONE* **4**, e5822 (2009).
52. Cirz, R. T. *et al.* Complete and SOS-Mediated Response of Staphylococcus aureus to the Antibiotic Ciprofloxacin. *Journal of Bacteriology* **189**, 531–539 (2007).
53. Mei, J.-M., Nourbakhsh, F., Ford, C. W. & Holden, D. W. Identification of Staphylococcus aureus virulence genes in a murine model of bacteraemia using signature-tagged mutagenesis. *Molecular Microbiology* **26**, 399–407 (1997).
54. Massière, F. & Badet-Denisot, M.-A. The mechanism of glutamine-dependent amidotransferases. *CMLS, Cell. Mol. Life Sci.* **54**, 205–222 (1998).
55. Qiu, X. *et al.* Crystal structure of Staphylococcus aureus tyrosyl-tRNA synthetase in complex with a class of potent and specific inhibitors. *Protein Science* **10**, 2008–2016 (2001).
56. Mikheyeva, I. V. *et al.* YpdA, a putative bacillithiol disulfide reductase, contributes to cellular redox homeostasis and virulence in Staphylococcus aureus. *Molecular Microbiology* **111**, 1039–1056 (2019).
